# Seasonal coordination of leaf hydraulics and gas exchange in a wintergreen fern

**DOI:** 10.1093/aobpla/plaa048

**Published:** 2020-09-11

**Authors:** Kyra A Prats, Craig R Brodersen

**Affiliations:** School of the Environment, Yale University, New Haven, CT, USA

**Keywords:** Fern, freeze–thaw, high light, photosynthesis, water relations, wintergreen, xylem transport

## Abstract

Wintergreen fern *Polystichum acrostichoides* has fronds that are photosynthetically active year-round, despite diurnal and seasonal changes in soil moisture, air temperature and light availability. This species can fix much of its annual carbon during periods when the deciduous canopy is open. Yet, remaining photosynthetically active year-round requires the maintenance of photosynthetic and hydraulic systems that are vulnerable to freeze–thaw cycles. We aimed to determine the anatomical and physiological strategies *P. acrostichoides* uses to maintain positive carbon gain, and the coordination between the hydraulic and photosynthetic systems. We found that the first night below 0 °C led to 25 % loss of conductivity (PLC) in stipes, suggesting that winter-induced embolism occurred. Maximum photosynthetic rate and chlorophyll fluorescence declined during winter but recovered by spring, despite PLC remaining high; the remaining hydraulic capacity was sufficient to supply the leaves with water. The onset of colder temperatures coincided with the development of a necrotic hinge zone at the stipe base, allowing fronds to overwinter lying prostrate and maintain a favourable leaf temperature. Our conductivity data show that the hinge zone did not affect leaf hydraulics because of the flexibility of the vasculature. Collectively, these strategies help *P. acrostichoides* to survive in northeastern forests.

## Teaser

Wintergreen ferns, such as *Polystichum acrostichoides*, retain their fronds throughout the year despite freezing temperatures and drastic seasonal changes in soil moisture and light intensity. We monitored photosynthetic capacity and the functional status of the vascular system for over a year and found that despite losing over 60 % of their water transport capacity from freeze–thaw induced cavitation, photosynthesis recovered in the spring. We found that localized, frost-triggered frond flexibility allows this species to lay flat on the ground and maintain warmer leaf temperatures, a process facilitated by highly flexible vascular bundles that bend without disrupting the water conducting pathway.

## Introduction

Temperate ferns are often associated with low-light habitats, such as the shaded understory beneath deciduous angiosperm-dominated canopies in the Northeastern USA (Minoletti and Boerner 1993). Fern species inhabiting the herbaceous understory play a critical role in forest ecosystems in terms of biodiversity, carbon gain and storage (George and Bazzaz 1999, 2014; Gilliam 2007, 2014). There are over 100 fern species in Connecticut alone (Connecticut Botanical Society 2015). While the majority of these fern species are deciduous, a few species retain their leaves and tolerate freezing winter temperatures, including the widespread *Polystichum acrostichoides* (Michx., Schott) (Tessier 2008, 2014). These species employ a wintergreen strategy—a type of evergreeness in which leaves produced in the spring survive for 1 year until they are replaced by the new cohort (Chabot and Hicks 1982). Adopting a wintergreen strategy thus extends the total length of photosynthetic activity per leaf compared with deciduous ferns (Chabot and Hicks 1982). This strategy allows photosynthesis to continue under higher light intensity conditions in the spring and fall when deciduous canopy leaves are absent (Noodén and Wagner Jr 1997; Forget *et al.* 2018). In a wintergreen *Dryopteris*, over 75 % of the total annual carbon gain occurs before canopy closure or after deciduous leaf senescence (Goldblum and Kwit 2012). Despite the respiratory cost, maintaining leaves over winter gives access to light during the open canopy period which promotes carbon storage and early growth in the following season (Chabot and Hicks 1982).

However, during canopy transition periods and over winter, temperature, precipitation and light quantity and quality change (Neufeld and Young 2003). Such fluctuations in environmental parameters influence leaf energy balance and net productivity (Neufeld and Young 2003). Exposure to the combination of low temperatures and high light intensity floods the photosynthetic apparatus with more light energy than it can use for carbon assimilation, causing the activation of photoprotective mechanisms such as the thermal dissipation of the excess light (Öquist and Huner 2003; Verhoeven 2014). In the absence of photoprotective mechanisms, the combination of low temperatures and high light can lead to low-temperature photoinhibition, which can damage the photosynthetic apparatus (Powles *et al.* 1983; Hetherington *et al.* 1989). Light intensity can fluctuate exponentially on both daily and seasonal timescales in the understory, and sunflecks have been shown to be important for carbon gain when the canopy is closed (Chazdon and Pearcy 1991). Wintergreen understory leaves must, therefore, tolerate the deep shade of the summer with periodic high intensity sunflecks, but also exposure to changing light quality and intensity coincident with low air temperatures when the canopy is open. It is not well understood how wintergreen ferns are capable of doing this, although some recent insights into photosynthesis and chlorophyll fluorescence have shown the influence of maintaining favourable leaf temperatures on carbon gain (Forget *et al.* 2018).

Compounding the effects of temperature and light on photosynthetic capacity is the vulnerability of the vascular system to embolism resulting from freeze–thaw cycles (Hammel 1967; Tyree and Zimmerman 2002). In the winter and early spring, freeze–thaw cycles make the xylem network vulnerable to embolism formation (Hammel 1967; Sucoff 1969; Zimmerman 1983; Sperry and Sullivan 1992; Hacke and Sperry 2001). Gas dissolved in the xylem sap comes out of solution during freezing, and bubbles expand to block xylem conduits once the sap thaws (Hammel 1967; Sucoff 1969; Zimmerman 1983; Sperry and Sullivan 1992; Hacke and Sperry 2001). Given the critical link between leaf hydraulic conductance and photosynthesis (Brodribb 2009), frequent winter freeze–thaw events should lead to significant declines in conductivity that impact photosynthesis (Brodersen *et al.* 2012). The temperature during such freezing events can determine the quantity of embolism, as more negative freezing temperatures can cause more embolisms (Lo Gullo and Salleo 1993; Pockman and Sperry 1997; Ball *et al.* 2006). Winter embolism can also result from frost or winter drought, whereby ice in frozen soil and plant organs blocks water transport (Mayr *et al.* 2002, 2003, 2019; Pittermann and Sperry 2003) and continued cuticular transpiration leads to water loss from the leaves (Duursma *et al.* 2019; Mayr *et al.* 2019). While there is a lack of understanding regarding winter embolism in angiosperms and gymnosperms (Mayr *et al.* 2019), the knowledge gap is all the more significant for wintergreen ferns. No previous studies have examined winter embolism in wintergreen ferns, and the mechanisms responsible for maintaining both photosynthetic capacity and hydraulic conductivity in response to freezing temperatures are unexplored.

As temperatures approach freezing, *P. acrostichoides* fronds undergo a leaf prostration process, whereby localized cell necrosis at the base of the stipe causes the once upright fronds to hinge to the ground (Noodén and Wagner Jr 1997; Forget *et al.* 2018) **[see Supporting Information—Movie S1]**. This unique morphological strategy occurs via necrosis of cortex cells in the stipe which leads to a highly localized destabilization of the structural integrity of the frond. Fronds hinge a few centimetres above the soil surface and lay in a prostrate position for the remainder of their life. This hinging is likely important for the winter survival of *P. acrostichoides* fronds. The repositioning of the leaf from upright to prostrate in the winter favourably influences leaf temperature, and also allows frond burial by the snow, thereby protecting the photosynthetic cells from frost damage (Givnish 1982; Tessier 2014; Forget *et al.* 2018). Together, these benefits likely allow the photosynthetic tissues to remain active and support the evergreen habit, but many specific physiological traits that enable this strategy to be successful are not well understood.

**Figure 1.**
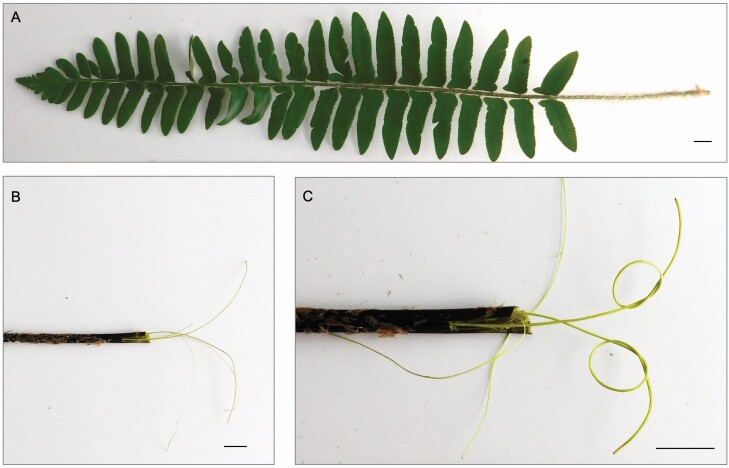
(A) A sterile *P. acrostichoides* frond. (B) Vascular bundles can be dislodged from the surrounding cortical tissue and extracted from the stipe (bundle extraction methods from Brodersen *et al.* 2012). (C) Vascular bundles are flexible and can be looped and bent without breaking. All scale bars are 1 cm.

Therefore, at least two factors may influence stipe hydraulic conductivity in *P. acrostichoides* on a seasonal timescale: freeze–thaw and winter embolism, and the physical bending of the stipe and vascular tissue therein. It has been found in many angiosperms and gymnosperms that conduit diameters in the 30–43 µm range resist bubble formation from freeze–thaw events (Davis *et al.* 1999; Pittermann and Sperry 2003). Conduit diameters above this threshold become vulnerable to bubble formation from freeze–thaw events at xylem pressures of −0.5 MPa (Davis *et al.* 1999; Pittermann and Sperry 2003). Yet at increasingly negative xylem sap pressures, the conduit diameter threshold to embolism vulnerability decreases, such that small diameter conduits become vulnerable (Pittermann and Sperry 2006). Therefore, both xylem pressure and conduit diameter interact to determine vulnerability to freeze–thaw embolism (Pittermann and Sperry 2006). We predicted mean tracheid diameter in *P. acrostichoides* stipes should fall below the 30–43 µm threshold to maintain a degree of conductivity after freeze–thaw events based on minimum temperatures experienced by fronds at our site. Unlike woody plants that feature secondary xylem, ferns only have primary xylem arranged in vascular bundles. Fern tracheids that comprise the xylem of these vascular bundles do not provide significant structural support for the plant (Niklas 1991; Rowe *et al.* 2004; McElwain 2011; Pittermann *et al.* 2011; Mahley *et al.* 2018), and the sole purpose of fern tracheids is water transport. Perhaps due to the minimal role that tracheids play in support, the vascular bundles of ferns are pliable (Fig. 1). The physical flexibility of vascular bundles is notable in the case of *P. acrostichoides* (Fig. 1) because it could allow the vascular system to remain functional even while the stipes bend and hinge to allow for leaf prostration. This hypothesis of the physiological importance of the vascular bundle flexibility has yet to be tested.

Here, we aimed to characterize the seasonal dynamics and coordination of the photosynthetic and vascular tissues in the wintergreen fern *P. acrostichoides*. In this way, we would be able to (i) determine the conditions that initiate the formation of the localized hinge zone in the stipe that leads to leaf prostration, (ii) characterize factors that influence stipe xylem hydraulic conductivity, (iii) examine the light environment and the effect of experimental shading on photosynthetic performance and (iv) determine the coordination between losses in hydraulic conductivity and photosynthesis or chlorophyll fluorescence. To achieve these goals, we measured net and maximum photosynthetic rates (on ambient and experimentally shaded ferns), chlorophyll fluorescence (*F*_*v*_*/F*_*m*_), hydraulic conductivity, xylem conduit diameter and flexural stiffness or bending modulus (*EI*) of hinge zones throughout the growing season.

## Methods

### Species and site description

*Polystichum acrostichoides* is a member of the *Polystichum sensu stricto* clade in the Dryopteridaceae (Little and Barrington 2003). It is a wintergreen fern that maintains fronds throughout the winter, with new fronds unfurling in the spring as the old fronds senesce (Greer and McCarthy 2000; Tessier 2014). Native to Eastern North America, *P. acrostichoides* is quite common in many forested sites (Greer and McCarthy 2000), including the study site. Because of its abundance, this species is important to the function of many forested ecosystems (Tessier 2008, 2014).

The field site was located at Yale Myers Forest in Eastford, Connecticut (41°57′31.7ʺN 72°07′37.5ʺW). The overstory community was primarily composed of *Acer rubrum* L., *Acer saccharum* and *Tsuga canadensis*, and was located on a slight, south facing slope adjacent to a lake. The field site was equipped with an understory weather station 1 m above the ground that monitored environmental parameters (CR300 and CR6 datalogger; Campbell Scientific, Logan, UT) using two soil moisture sensors (Campbell Scientific; #CS650), a temperature and relative humidity sensor (Campbell Scientific; #CS215), a precipitation gauge (Campbell Scientific; #TE525), and three photosynthetic photon flux density (PPFD) sensors (LiCor Inc, Lincoln, NE; #LI190). It is important to note that air temperature measured by our weather station at a height of 1 m can underestimate ground level temperature by a few degrees. Sensors logged data hourly for most of the duration of the experiment, as the weather station was installed two months into the physiological measurement sampling regime in July 2017. We measured minimum air temperatures (*T*_*min*_) recorded the night prior to each field sampling date to determine the relationship between temperature and physiology, hydraulics and hinge formation. Coincident weather data from a nearby weather station (Bradley International Airport Station, 40 km) was used to determine *T*_min_ prior to July 2017 (Weather Underground and Company 2019). We found good agreement between *T*_min_ values obtained from the local weather station and our micrometeorological station at the field site **[see ****Supporting Information—Fig. S1****]**. Our site was also equipped with two time-lapse cameras (TimelapseCam Pro, Wingscapes, Calera, AL) that captured one photograph at 11:00 AM every day to track the phenology of the fern population. We conducted field work and performed the physiological and hydraulics measurements approximately once per month between April 2017 and May 2018. This sampling frequency and duration allowed us to monitor the senescence of the 2016 cohort of fronds (four individuals) and the entire lifespan of the 2017 cohort of fronds (six individuals).

### Shade structures

In September 2017, two, 1 m^3^ shade structures were installed at the field site over four individuals of *P. acrostichoides* from the 2017 cohort, two of which had been tagged and previously measured. The shade structures were constructed from 0.75″ PVC pipe and wrapped with shade cloth to extend the low light levels received by these ferns into the winter and early spring, thereby simulating understory light intensity conditions of summer. The shade cloth did not have any influence on the spectral quality of the light. One of the PPFD sensors from the weather station was installed under a shade structure to compare the light conditions under the structure with those in ambient conditions. We compared the photosynthetic rates of fronds from under the shade structure to fronds of separate nearby plants (<5 m) that were in ambient conditions to determine the effect of shading throughout the winter on photosynthesis.

### Field data collection: water potentials

We measured whole frond water potential with a Scholander pressure chamber (Model 600, PMS Instruments, Albany, OR). Sterile fronds were covered with mylar coated plastic bags for 30 min to stop transpiration and allow water potentials of fronds to equilibrate (Tyree and Zimmerman 2002; Brodersen *et al.* 2012). Once equilibrated, we cut the base of the stipes and measured the xylem water potential by pressurizing fronds within the chamber. Additionally, we measured soil moisture (volumetric water content, %) in three locations 30 cm from the base of six ferns (Theta Probe ML2x; HH2 moisture metre, Delta-T Devices, Cambridge,UK).

### Field data collection: LI-6400 photosynthesis and fluorescence

Photosynthesis and chlorophyll fluorescence were measured between 9:00 AM and 11:00 AM to avoid afternoon depression of photosynthesis using a LI-6400 equipped with a Leaf Chamber Fluorometer (6400–40, Li-Cor, Lincoln, NE). Portions of sterile fronds halfway up the length of the rachis from six *P. acrostichoides* individuals were dark adapted for 1 h using dark adapting clips (9964-091, Li-Cor). We then performed light response curves (LRCs) on non-dark adapted portions halfway up the length of the rachis, but on the opposite side, of the same six fronds. These LRCs were conducted to find the rate of dark respiration, the maximum rate of photosynthetic assimilation (*A*_max_) and the light intensity at which *A*_max_ occurs. We began our LRCs at an intensity of 0 µmol m^−2^ s^−1^ to capture dark respiration, and the photosynthetic assimilation values were logged after the frond achieved stability; we repeated this process at light intensities of 50, 100, 200, 400, 600, 800 and 1000 µmol m^−2^ s^−1^. The fluorescence head was used to measured baseline *F*_0_ before sending a quick and intense flash of saturating light to the frond to measure *F*_*m*_. The *F*_*v*_*/F*_*m*_ ratio was then calculated, representing a measure of efficiency of PSII (Krause and Weis 1991; Baker 2008; Murchie and Lawson 2013; Jones 2014). Fronds were excised at the base of the stipe as close to the rhizome as possible, placed in a plastic bag with moist paper towels, and brought to the lab for further analyses.

### Modelling LRCs

Data from LRCs were modelled in Excel using the tool developed by Lobo *et al.* (2013) using the Baly (1935) model (Baly 1935; Lobo *et al.* 2013) to find the maximum photosynthetic rates (*A*_max_) for each date that gas exchange was sampled. The Baly (1935) model was applied:


$$\begin{array}{l} A_{N}=\left[\frac{{\;\Phi \;}_{Io}\times \;I\;\times \;A_{max}}{{\;\Phi \;}_{Io}\times \;I+\;A_{max}}\right]-\;R_{D} \end{array}$$ (1)

where *A*_N_ is the net photosynthesis rate in µmol (CO_2_) m^−2^ s^−1^, Φ _*Io*_ is the quantum yield at *I* = 0 in µmol (CO_2_) µmol^−1^ (photons), *I* is the photosynthetic photon flux density (PPFD) in µmol (photons) m^−2^ s^−1^, *A*_*max*_ is the maximum gross photosynthesis rate in µmol (CO_2_) m^−2^ s^−1^ and *R*_*D*_ is the dark respiration rate in µmol (CO_2_) m^−2^ s^−1^ (Baly 1935; Lobo *et al.* 2013). Model inputs for each date of sampling comprised the mean assimilation values (*A*) values from our measured LRCs for all *P. acrostichoides* ferns. Predicted *A* values from the model were also used to create a net carbon assimilation value for each hourly light intensity value (PPFD) recorded by the weather station on sampling dates, resulting in a diurnal net carbon assimilation model.

### Hydraulics

We characterized the hydraulic conductivity of the stipe xylem by measuring the flow rate in a given length of stem segment for a given pressure gradient using the following equation:


$$\begin{array}{l} K=F\frac{L}{\Delta P} \end{array}$$ (2)

where *K* is conductivity in mmol m s^−1^ MPa^−1^, *F* is the flow rate in mmol s^−1^, *L* is the length of the stipe segment (in m) and *ΔP* is the pressure gradient in MPa (Tyree and Zimmerman 2002). To measure stipe native conductivity (*K*_native_), the base of the stipes were cut underwater twice on each end into segments of 10 cm in length. These stipe segments were connected to hydraulic tubing filled with a filtered and degassed 20 mM KCl solution. Background flows were recorded before and after measurements of pressurized flow using a liquid flow sensor (Model SLI, Sensirion, Switzerland). Stipes were submerged in water and degassed overnight in a filtered 20 mM KCl solution connected to a vacuum valve to remove embolism for measuring maximum conductivity (*K*_max_). We calculated percent loss of conductivity (PLC) as 100 × [1 − (*K*_native_/*K*_max_)] (Tyree and Zimmerman 2002). We collected fronds in November before and after the first frost that were used exclusively for these hydraulics measurements; otherwise hydraulics measurements were conducted on the same fronds that were measured in the field for photosynthetic parameters. Stipes were preserved and freehand cross-sections were prepared with a razor blade for transverse light microscopy (Olympus BX60, Olympus America, Center Valley, PA, USA). Thin cross-sections were mounted in water under a cover slip and then photographed at 20×. The tracheid diameter distribution was characterized by measuring 185 conduits across all individuals using ImageJ (v2.0, U.S. National Institutes of Health, Bethesda, MD, USA).

### Single-conduit air-seeding

To determine the vulnerability of the tracheids to drought-induced embolism we measured the air-seeding threshold using the single-capillary method as described in Brodersen *et al.* 2014 for ferns. Briefly, six stipes were collected and trimmed to a length of 4 cm with a razor blade to expose the transverse surface of the vascular tissue; stipes were mounted in a multi-position vice (Panavise model 201, Medford, OR, USA). Glass capillary tubes (WPI, 1B150-4; WPI Inc., Sarasota, FL, USA) were pulled to a tip diameter of ~15 µm (Vertical Micropipette Puller, model P-30; Sutter Instrument, Novato, CA, USA) and inserted into the open end of a tracheid using a micromanipulator and a stereo microscope (Leica S6 stereo microscope, Wetzlar, Germany). The proximal end of the stipe was sealed with cyanoacrylic glue (Loctite 409; Loctite, Düsseldorf, Germany) and a hardening accelerant (Loctite 712) to secure the capillary tube and prevent leaks. The distal end was then submerged in water. The glass capillary tube was then connected to a 1 m length of PEEK tubing (51085K48; McMaster-CARR, Princeton, NJ, USA) with its terminus mounted in a Scholander pressure chamber (#1505; PMS Instruments, Corvallis, OR, USA). Pressure in the chamber and tubing was increased slowly until a steady stream of bubbles could be seen emerging from the distal end of the stipe submerged in water. This positive pressure was recorded as the air-seeding threshold, and equal to but opposite sign of the water potential that would lead to air entry in the xylem *in situ*.

### Biomechanics of the hinge zone

After measuring hydraulic conductivity at our sampling frequency of approximately once a month, we used the same stipe segments—trimmed down to 2-cm in length—to perform three-point bending tests using an Instron single-column tabletop testing system (Instron 5943, Norwood, MA) to compare the flexural stiffness (*EI*) of hinged and unhinged stipes. We measured the radius of each stipe at the midway point (1 cm) using a calliper to calculate the second moment of area (*I*); *I* is a characteristic of the arrangement and the dimensions of the object, and for a solid cylindrical beam, such as a petiole, is given by


$$\begin{array}{l} I=\;\;\;\frac{\pi r^{4}}{4} \end{array}$$ (3)

where *r* is the radius of the cylinder. The value of *I* is necessary for the calculation of *EI*. The stipe segments were placed on the lower two supports—with 1.5 cm of distance between them—with the frond’s abaxial side (with three small bundles) facing up and the adaxial side (two larger bundles) facing down. We point-loaded each stipe in the middle of the segment, with the abaxial side receiving compressive stress and the adaxial side receiving tensile stress. The Instron applied a point-loaded force to bend the stipe, moving at a rate of 10 mm min^−1^. Force (*F*) divided by cross-sectional area (*A*) provides a measure of stress that is applied to an object. Strain, a measure of how that object reacts to stress, is calculated from the change in length (extension) divided by the original length (Vogel 2013). The slope of this graph is the Young’s modulus of elasticity (*E*), which is calculated as


$$\begin{array}{l} E=\;\frac{\sigma }{\varepsilon } \end{array}$$ (4)

where σ is stress and ε is strain and *E* is measured in dimensions of force divided by area. The *EI* is, intuitively, the product of the *E* and *I*, which was calculated for hinged and unhinged stipes. All measurements across the seasons of hinged and unhinged stipes were combined into these two categories for statistical analysis.

We used transverse light microscopy to image cross-sections of hinged and unhinged sections of stipes. Samples were prepared using a GSL1 microtome (Zurich, Switzerland), mounted on slides with water, and immediately imaged on an Olympus compound microscope (BX60, Olympus America, Center Valley, PA, USA) at 4× magnification. Images were taken with a Canon 6D DSLR camera and stitched together using pairwise stitching in ImageJ (Preibisch *et al.* 2009).

### Leaf temperature and prostration

To determine the effect of leaf position (prostrate or upright) on leaf temperature, we experimentally manipulated leaves in the field and then recorded leaf temperature similar to the investigations carried out by Forget *et al.* (2018). On an April morning (between 9:00 AM and 11:00 AM) in 2018, we randomly selected 20 prostrate fronds from individuals in the same sunny area and measured leaf temperature using an infrared, single dot laser thermometer at a distance of 10 cm from the lamina that uses wavelengths of 8–14 µm with an accuracy of ±1.5 % (Fluke Instruments at W.W. Grainger Inc, Lake Forest, IL, USA). Leaf temperature was measured in the middle lamina on two pinnae halfway up the length of each rachis. Ten of the fronds were artificially propped up to a 45˚ angle from horizontal using two crossed metal flagging stakes (Uline, Pleasant Prairie, WI, USA) to create a cradle for the fronds to lie in. We let upright fronds equilibrate for 30 min before measuring leaf temperature again on all 20 fronds, 10 prostrate and 10 upright.

### Statistics

For each sampling date, measurement values for each parameter from all measured ferns were pooled into a mean value. We ran univariate linear regression models of environmental parameters and physiological parameters using the lm() function in R. Specifically, we ran linear regression models of *F*_*v*_*/F*_*m*_ and *T*_*min*_ as predictors of *A*_*max*_. To determine the factors influencing PLC, we ran linear regression models with water potential, soil moisture and *T*_*min*_ as separate predictor variables of PLC. We performed a paired *t*-test to analyse differences in *A*_*max*_ between shade-treated and ambient plants. Additionally, we performed Welch’s two-sample *t*-tests to test to identify biomechanical differences between hinged and unhinged stipes, as well as for leaf temperature between propped fronds and prostrate fronds. All statistics were conducted in R version 3.6.2 (R Foundation for Statistical Computing, Vienna).

## Results

### Phenology and environmental parameters

**Figure 2.**
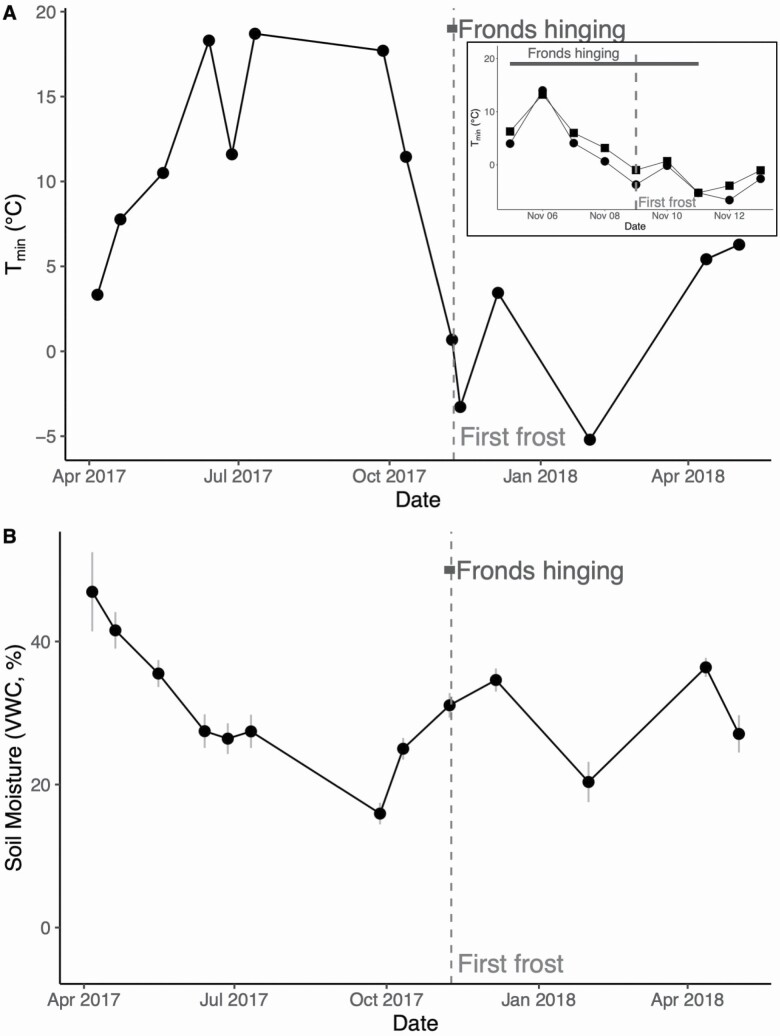
(A) Minimum night-time air temperature (*T*_*min*_) across the seasons. Inset figure shows minimum night-time air temperature (circles) and soil temperature (squares) for the period of 5th to 13th November during frond hinging and the first frost. (B) Soil moisture as volumetric water content (VWC) across the seasons. The light grey vertical dashed lines in all figures indicate the timing of the first frost on 9 November 2017, while the dark grey horizontal lines represent the timing of frond hinging between 5th November and 11th November, as seen from time-lapse cameras. Open circles represent fronds from the 2016 cohort, while closed circles show data from the 2017 cohort, and grey bars in (B) represent standard error of the mean.

Time-lapse images taken daily of fronds revealed their phenology, in particular the timing of frond hinging. Fronds began to hinge on 5 November 2017, with the majority of fronds hinging fully within a 24-h period on 11 November 2017 **[see Supporting Information—Movie S1]**. From our temperature data (Fig. 2A), we know that 9 November was the first day the fronds experienced a minimum night-time air temperature (*T*_min_) below freezing, suggesting that the timing of fronds hinging coincided with the first freezing event. Air *T*_min_ (circles in Fig. 2A inset) and soil *T*_min_ (squares in Fig. 2A inset) decline during the period of frond hinging before dipping below 0 °C on 9 November. While we observed predictable seasonal patterns from our measures of *T*_min_ (Fig. 2A), seasonal soil moisture did not reflect predictable seasonal patterns (Fig. 2B). There were no abrupt changes in soil moisture around the time of frond hinging.

### Photosynthesis, fluorescence and LRCs

**Figure 3.**
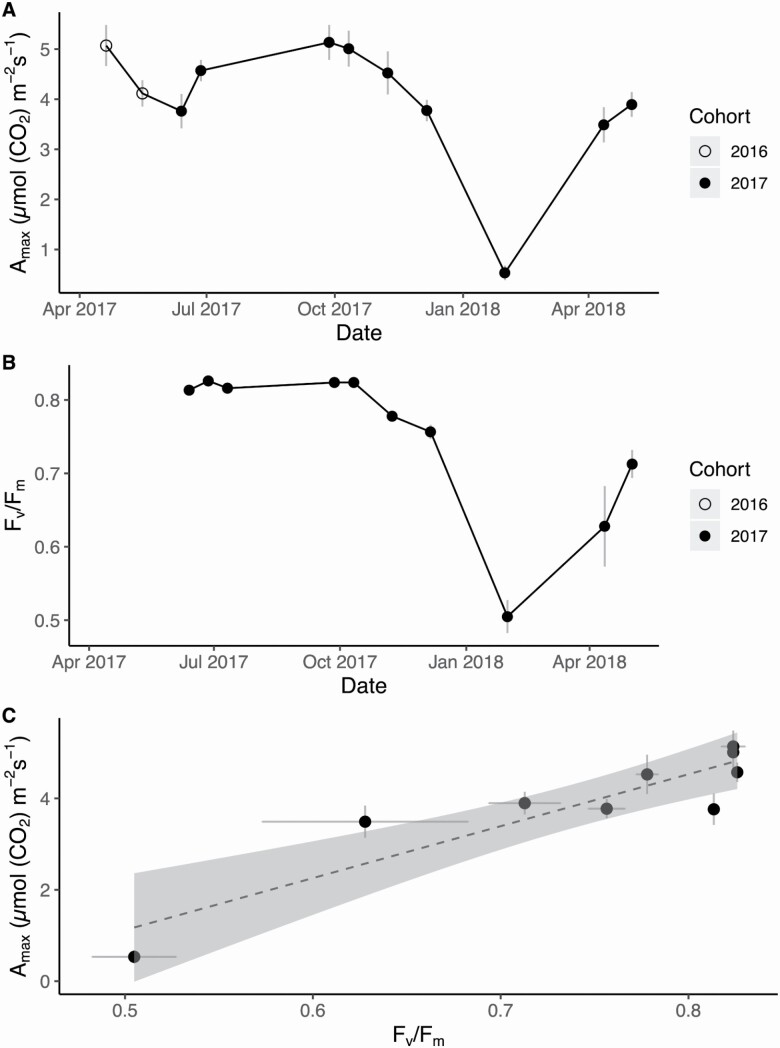
(A) Mean maximum photosynthetic rate (*A*_*max*_) measured for both cohorts and (B) mean photosynthetic efficiency (*F*_*v*_*/F*_*m*_) measured for the 2017 cohort show similar patterns of winter decline and spring recovery over the course of the year. Open circles represent measurements on fronds from the 2016 cohort, while closed circles are fronds from the 2017 cohort. (C) The linear regression model of mean *A*_*max*_ and *F*_*v*_*/F*_*m*_ was significant and positive (Adjusted R-squared = 0.73, F = 23.1, *P* = 0.0019). All values were generated from fronds that belonged to the 2017 cohort. The grey shaded area around the dashed regression line represents the 95 % confidence interval. Grey bars in (A–C) represent standard error of the mean.

We determined average *A*_max_ from the LRC models (Fig. 3A), and then explored their seasonal dynamics and relationship to *F*_*v*_*/F*_*m*_ (Fig. 3B). Mean *A*_max_ peaked in the spring and stayed high through the summer and fall of 2017, and then declined from November to a minimum in January 2018. *A*_max_ recovered to previous season values in the spring months of 2018 (Fig. 3A). Similarly, *F*_*v*_*/F*_*m*_ remained stable and within an unstressed range (between 0.75 and 0.85) until the January sampling date, when the mean *F*_*v*_*/F*_*m*_ declined to 0.50 (Fig. 3B). Mean *F*_*v*_*/F*_*m*_ recovered in the spring of 2018 to 86 % of the maximum recorded the previous year. Although the mean *A*_max_ of shade-treated ferns was lower than that of the ambient ferns, the differences in *A*_max_ were not significantly different when compared with control plants (paired *t*-test: *T* = 1.8, DF = 5, *P*-value = 0.13) **[see ****Supporting Information—Fig. S2****]**.

**Figure 4.**
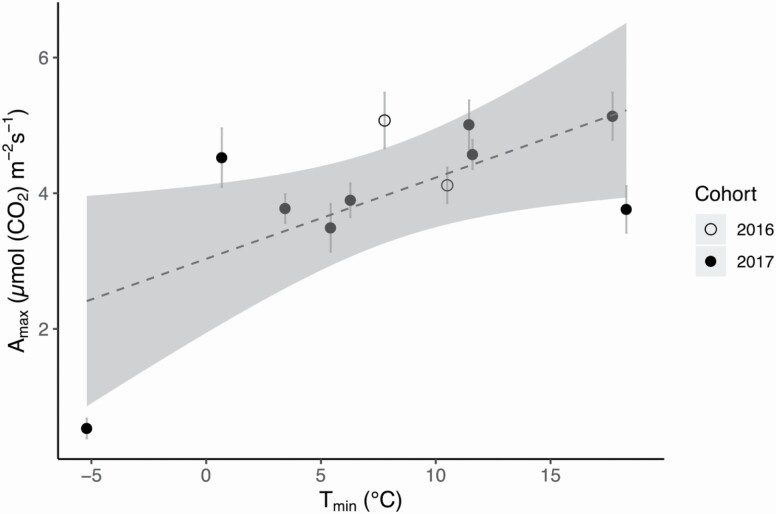
Linear relationship between mean maximum photosynthetic rate (*A*_*max*_) and minimum night time temperature (*T*_*min*_) (Adjusted R-squared = 0.36, F = 6.7, *P* = 0.029). Open circles represent measurements on fronds from the 2016 cohort, while closed circles show data from fronds from the 2017 cohort. The grey shaded area around the dashed regression line represents the 95 % confidence interval, and grey bars represent standard error of the mean.

We found that mean *F*_*v*_*/F*_*m*_ was significantly and positively related to both measured and modelled mean *A*_max_ (linear regression models: measured adjusted *R*^2^ = 0.73, F = 23.1, *P* = 0.0019, Fig. 3C; modelled adjusted *R*^2^ = 0.81, *F* = 34.1, *P* = 0.00064, **see ****Supporting Information—Fig. S3**). Maximum PPFD of the sampling day was not a significant predictor of measured mean *A*_max_ (linear regression model: Adjusted R-squared = −0.02, F = 0.89, *P* = 0.39, **see ****Supporting Information—Fig S4**). However, measured and modelled mean *A*_*max*_ both had significant positive relationships with *T*_*min*_ (linear regression models: measured adjusted *R*^2^ = 0.36, *F* = 6.7, *P* = 0.029, Fig. 4; modelled adjusted *R*^2^ = 0.44, *F* = 8.9, *P* = 0.015, **see ****Supporting Information—Fig. S5**). Higher *T*_min_ values recorded the night before the sampling were associated with higher modelled and measured *A*_max_ values. Frond water potential was not a good predictor of measured mean *A*_max_ (linear regression model: adjusted *R*^2^ = 0.23, F = 4.0, *P* = 0.07, **see ****Supporting Information—Fig. S6**) within the range of water potentials experienced by plants at our site during the sampling period.

**Figure 5.**
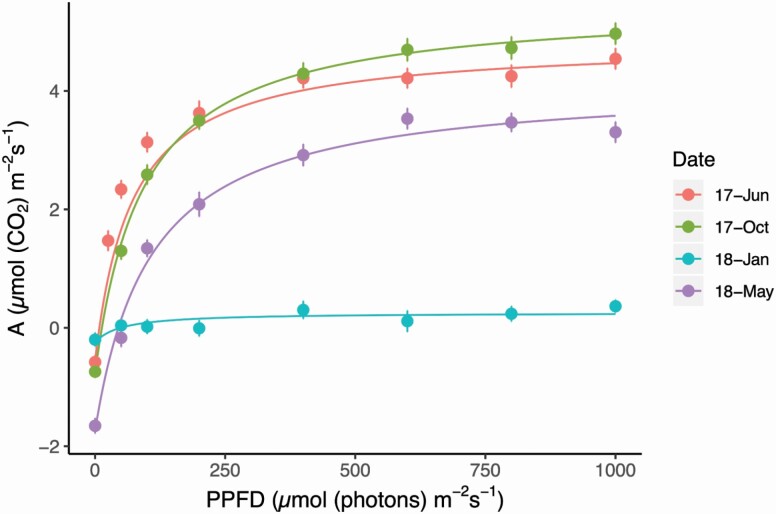
Seasonal response of photosynthesis (*A*) to increasing light intensity (PPFD) at four representative months (June 2017-summer; October 2017-fall, January 2018-winter and May 2018-spring) depict measured photosynthetic values (circles with standard error bars) and modelled curves (lines). Full LRC parameters can be found in Table 1.

Average LRCs were modelled in Excel (Baly 1935; Lobo *et al.* 2013) to determine the maximum photosynthetic rate (*A*_max_), light compensation point (*I*_comp_), light saturation point (*I*_max_), dark respiration rate (*R*_D_), quantum yield at irradiance of zero (Φ _*Io*_) and the net photosynthesis at the light saturation point (*A*_N(*I*max)_) for each sampling date (Table 1). Representative modelled LRCs for June 2017, October 2017, January 2018 and May 2018 show changing curve shapes and *R*_*D*_ across the seasons (Fig. 5). Particularly, *R*_D_ was highest (−1.7 µmol (CO_2_) m^−2^ s^−1^) when fronds were senescing in May 2018, which also resulted in a higher *I*_comp_ (44.6 µmol (photons) m^−2^ s^−1^) compared with the mean annual *I*_comp_ of 17.5 µmol (photons) m^−2^ s^−1^. The LRC from January 2018 was flat compared with the other curves, and only reached an *I*_*max*_ of 65 µmol (photons) m^−2^ s^−1^; comparatively, the annual mean *I*_*max*_ was 428.9 µmol (photons) m^−2^ s^−1^. Additionally, mean *A*_max_ in January was marginally positive at only 0.5 µmol (CO_2_) m^−2^ s^−1^. We also modelled LRCs for the ferns under the shade treatment to compare to those under ambient conditions **[see ****Supporting Information—Table S1****]**; in general, the shade-treated ferns had lower *A*_max_ than the ferns in ambient conditions, although the differences were not significant. A full comparison of modelled parameters from shade-treatment and ambient LRCs can be found in **Supporting Information—Table S1**.

**Table 1.** Parameters and variable estimates from LRC models; *A*_*max*_ is the maximum gross photosynthetic rate in µmol (CO_2_) m^−2^ s^−1^, *I*_*comp*_ is the light compensation point in µmol (photons) m^−2^ s^−1^, *I*_*max*_ is the light saturation point in µmol (photons) m^−2^ s^−1^, *R*_*D*_ is the dark respiration rate in µmol (CO_2_) m^−2^ s^−1^, Φ _*Io*_ is the quantum yield at *I* = 0 in µmol (CO_2_) µmol^−1^ (photons) and *A*_N(*I*max)_ is the net photosynthetic rate at the light saturation point in µmol (CO_2_) m^−2^ s^−1^ (Baly 1935; Lobo *et al.* 2013). Data for each date of sampling comprised the mean LRC values (*A*_*N*_) for four to six *P. acrostichoides* ferns. Mean values across the whole year are presented in the last row, with standard deviation in parentheses.

Date of sampling	*A*_*max*_	*I*_*comp*_	*I*_*max*_	*R*_*D*_	Φ_*Io*_	*A*_N(*I*max)_
20 April 2017^**†**^	6.4	17.5	605.0	0.8	0.0548	5.2
16 May 2017^**†**^	5.0	11.3	468.0	0.6	0.0556	3.6
13 June 2017	5.0	24.2	439.0	1.2	0.0640	3.1
27 June 2017	5.4	10.0	485.0	0.5	0.0750	4.0
27 Sept. 2017	6.0	9.2	535.0	0.5	0.0635	4.6
11 Oct. 2017	6.2	12.9	541.0	0.8	0.0673	4.6
8 Nov. 2017	4.8	3.9	387.0	0.3	0.0761	3.8
6. Dec.2017	3.7	0.0	337.0	0.0	0.0553	3.1
31 Jan. 2018	0.5	48.7	65.0	0.2	0.0100	0.0
12 April 2018	3.6	10.2	301.0	0.6	0.0682	2.5
2 May 2018	5.9	44.6	555.0	1.7	0.0551	3.2
**Annual mean**	4.8 (1.7)	17.5 (15.8)	428.9 (158.1)	0.65 (0.47)	0.0573 (0.0171)	3.4 (1.4)

^**†**^2016 cohort of fronds. All remaining dates sampled from the 2017 cohort of fronds.

### Loss of hydraulic function

**Figure 6.**
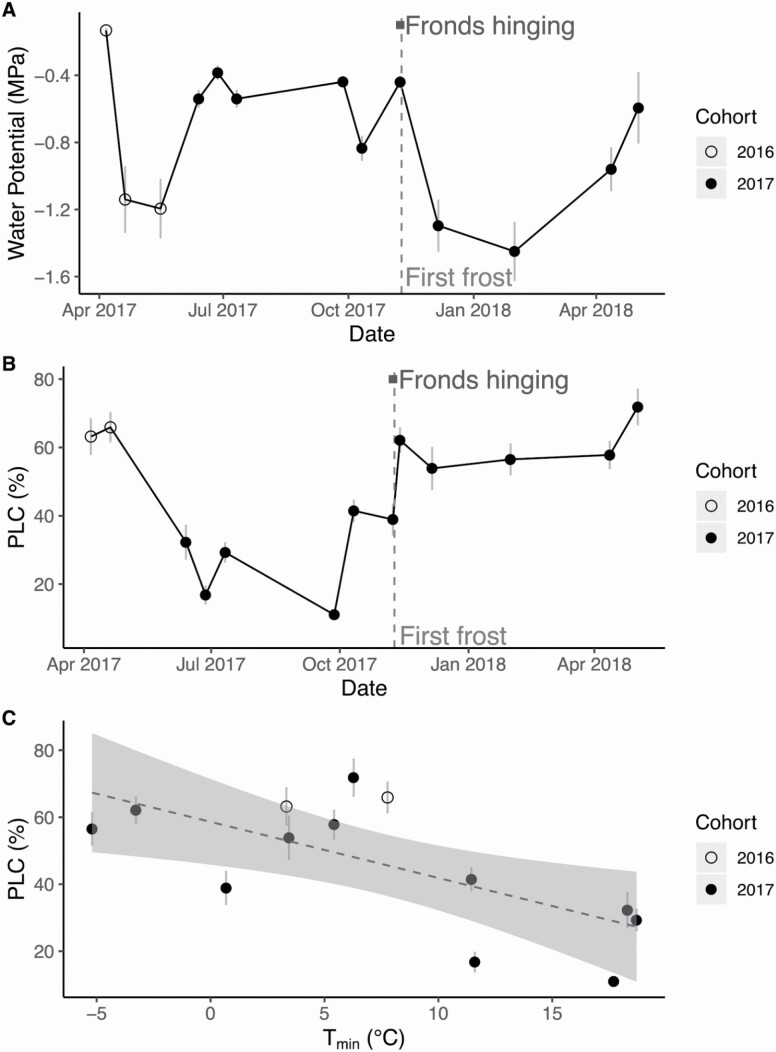
(A) Mean frond water potential and (B) mean percent loss of hydraulic conductivity (PLC) over time. The light grey vertical dashed line in (A) and (B) indicates the timing of the first frost on 9 November 2017, while the dark grey horizontal line represents the timing of frond hinging between 5th November and 11th November, as seen from time-lapse cameras. (C) The linear regression model of mean PLC and *T*_*min*_ was significant and negative (Adjusted R-squared = 0.41, F = 9.3, *P* = 0.011). The grey shaded area around the dashed regression line represents the 95 % confidence interval. Open circles in (A–C) represent measurements on fronds from the 2016 cohort, while closed circles are fronds from the 2017 cohort, and grey bars represent standard error of the mean.

Frond water potentials were least negative in the early spring and summer, and most negative in the winter (Fig. 6A). Mean PLC data indicated that stipes carried some embolism throughout the growing season, with a sharp increase in PLC immediately following the first frost event on the morning of 9 November 2017 (Fig. 6B). Notably, the spike in PLC following the frost event occurred when soil water potential values were high (−0.4 MPa; Fig. 5A). Mean PLC values were high in fronds from the 2016 cohort that had overwintered (open symbols in Fig. 6B). Mean PLC increased by 25 % between 8 and 13 November 2017, sampling dates that were before and after the first frost, exposure to freezing temperatures, and subsequent daytime thawing. Mean PLC values remained high (~60 %) into the spring of 2018 and did not recover.

In terms of the factors influencing PLC, there was no significant relationship between frond water potential and PLC within the range of water potentials measured in this study (adjusted *R*^2^ = 0.09, *F* = 2.1, *P* = 0.176, **see ****Supporting Information—Fig. S7A**). There was a weak but statistically significant positive relationship between soil moisture and PLC (adjusted *R*^2^ = 0.31, F = 5.8, *P* = 0.036, **see ****Supporting Information—Fig. S7B**). We then compared *T*_min_ with the PLC of the fronds and found a significant negative relationship (adjusted *R*^2^ = 0.41, F = 9.3, *P* = 0.011, Fig. 6C). Out of all of the environmental variables tested, *T*_min_ was the best predictor of PLC.

After confirming this link between PLC and *T*_min_, we explored potential underlying mechanisms, including freeze-thaw induced cavitation. Mean stipe tracheid diameter was 12.8 ± 0.47 µm **[see ****Supporting Information—Fig. S8****]**, which is below the predicted 30–43 µm critical threshold for severe vulnerability to freeze–thaw embolism in conifers (Davis *et al.* 1999; Pittermann and Sperry 2003). Out of 185 tracheids measured approximately 1 % were greater than 30 µm in diameter. We determined that the mean air-seeding threshold for *P. acrostichoides* was −3.19 MPa (±0.75SD).

### The hinge zone—biomechanics, anatomy, hydraulics and leaf temperature

**Figure 7.**
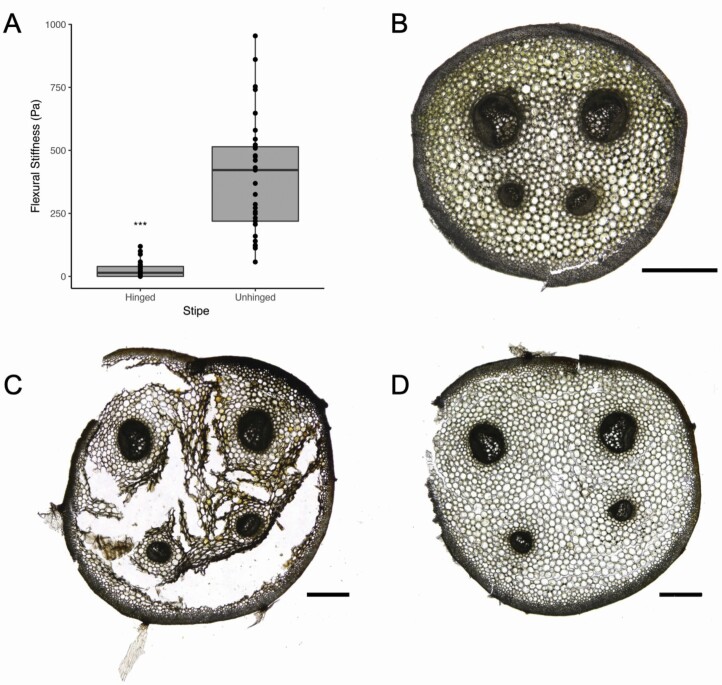
(A) The flexural stiffness of hinged and unhinged stipes was significantly different (Welch two-sample *t*-test: T = -9.47, DF = 33.02, *P*-value = 6.23e-13). The mean flexural stiffness of hinged stipes was 25 Pa, while the mean flexural stiffness of unhinged stipes was 402 Pa. All measurements across the seasons of hinged and unhinged stipes were combined into these two categories. (B) A cross-section image of an unhinged stipe. (C) A cross-section image of a severely necrotized stipe base within the hinge zone shows the friable nature of the cortex, with vascular bundles preserved. (D) This cross-section image was taken a few centimetres above the hinge zone on the same stipe as (C), which appears similar to the unhinged stipe in (B). All images in (B–D) are transverse light microscopy, with a scale bar of 50 µm.

The flexural stiffness or bending modulus (*EI*) of unhinged control stipes was significantly higher than hinged stipes with necrosis (Welch two-sample *t*-test: T= −9.47, DF = 33.02, *P*-value = 6.23e−13, Fig. 7A). Overall, unhinged stipes had a mean *EI* of 402Pa (±227 SD) and exhibited more variability than that of hinged stipes, which was uniformly low at a mean of 25 Pa (± 30 SD).

Transverse light microscopy images of the stipe within the hinge zone revealed substantial necrosis within the cortex (Fig. 7C), yet the vascular bundles were qualitatively similar to those within unhinged stipes (Fig. 7B). In some cases, severe cortex necrosis led to disintegration of the cells surrounding the vascular bundles (Fig. 7C). Marked differences in the perimeter of the hinged and unhinged stipes were evident. In hinged stipes, the cortical tissue 1.5 cm above the hinge zone was not necrotic (Fig. 7D).

To determine the effect of leaf angle and the role of the hinge zone on frond temperature, we measured frond temperature on prostrate and artificially upright fronds. There was no difference in leaf temperature before the experiment while all fronds were prostrate (Welch two-sample *t*-test: T = 0.13, DF = 13.1, *P* = 0.90). Mean leaf temperature of the 10 fronds that remained prostrate was significantly warmer at 14.7 °C than the temperature of the fronds that were propped up, at 12.5 °C (Welch two-sample *t*-test: T = 3.06, DF = 16.3, *P* = 0.007; **see ****Supporting Information—Fig. S9**).

### Carbon assimilation

**Figure 8.**
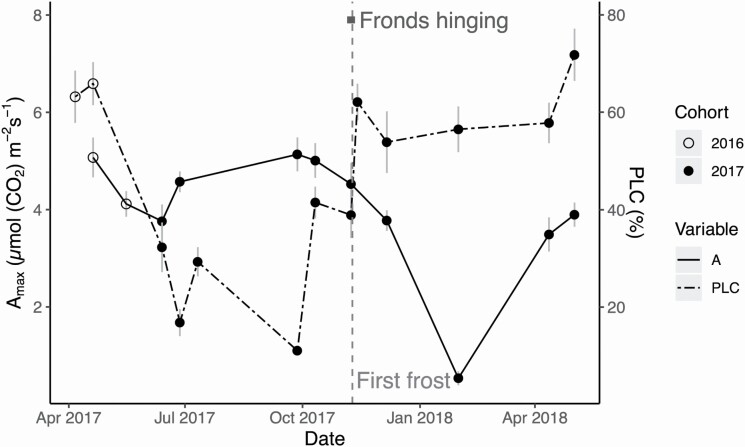
The coordination between *A*_*max*_ (solid line) and PLC (dash-dot line) across the seasons. Despite PLC remaining high in the spring of 2018, *A*_*max*_ recovers to pre-winter levels. The light grey vertical dashed line is the timing of the first frost on 9 November 2017, while the dark grey horizontal line represents the timing of frond hinging between 5th November and 11th November, as seen from time-lapse cameras. Open circles represent measurements on fronds from the 2016 cohort, while closed circles are fronds from the 2017 cohort. Bars represent standard error of the mean.

When comparing the photosynthetic performance to the hydraulic performance, we observed *A*_max_ and *F*_*v*_*/F*_*m*_ recover to pre-winter values in the spring, despite the PLC remaining permanently high (Fig. 8). Using the LRC models, we generated diurnal carbon assimilation curves for each sampling day **[see ****Supporting Information—Fig. S10A–J****]**. Net carbon assimilation was negative only on the January sampling date **[see ****Supporting Information—Fig. S10G and J****]**. These data show that *P. acrostichoides* can continue to assimilate carbon outside of the growing season (e.g. November and April) when the canopy is open.

## Discussion

Our data show that *T*_min_ is the most influential environmental factor in determining net carbon gain for *P. acrostichoides* within our study system, and the lost conductivity from winter freeze–thaw embolism did not prevent the recovery of photosynthesis in the spring. Minimum temperatures have important implications for both photosynthesis and xylem function, but also in triggering localized stipe necrosis and frond hinging.

### Photosynthetic performance

The photosynthetic performance of ferns under the experimental shade structures did not differ from that of ambient control ferns in a statistically meaningful way. This finding suggests that either photosynthetic response to sun and shade is fairly conserved in this species, or the difference in light intensity imposed by the structures was not great enough to elicit a photosynthetic response. Mean *A*_max_ and *F*_*v*_*/F*_*m*_ recovered to pre-winter values in the spring after exposure to below freezing temperatures of winter. The photosynthetic performance (in terms of *A*_max_ and *F*_*v*_*/F*_*m*_) was driven by *T*_min_, as the strong positive correlation suggests. Our findings in a wintergreen fern are consistent with results from over-wintering angiosperms that experience photoprotection or photoinhibition (Farage and Long 1991; Long and Humphries 1994; Farage *et al.* 2006). Low temperatures, particularly near or below-freezing, slow or halt the enzymatic processes of photosynthesis, from the electron transport chain to the functioning of Rubisco (Öquist 1983). The combination of low temperatures and high light intensity can overwhelm the photosystems with excess light, which can activate photoprotective mechanisms such as thermal dissipation of the excess energy (Öquist and Huner 2003; Verhoeven 2014). In the absence of photoprotection, low temperatures and high light can potentially lead to low-temperature photoinhibition, which is damaging for the photosynthetic systems (Powles *et al.* 1983; Hetherington *et al.* 1989). While photoprotection via increased carotenoid concentrations and thermal dissipation of light energy has been documented in tropical ferns under water stress (Tausz *et al.* 2001), we do not know of any published studies on this topic in wintergreen ferns. Because both photoprotection and photoinhibition can cause declines in *F*_*v*_*/F*_*m*_ values in winter (Öquist and Huner 2003; Verhoeven 2014), we cannot distinguish between the two in the current study.

The importance of temperature in governing photosynthetic performance is supported by the fact that *T*_min_ was a better predictor of *A*_max_ (Fig. 4) than PPFD **[see ****Supporting Information—Fig. S4****]** or water potentials **[see ****Supporting Information—Fig. S5****]**. The results from our LRCs also show that the features of the curves (i.e. *I*_comp_, *I*_max_, Φ _*Io*_) are relatively consistent throughout the year as PPFD changes with canopy coverage, but that our curve from the middle of winter was severely impacted by below-freezing temperatures. While significant attention has been devoted to studies of freezing response in angiosperms and gymnosperms (Farage and Long 1991; Long and Humphries 1994; Farage *et al.* 2006), wintergreen ferns have been excluded from such analyses.

We found a different trend in seasonal carbon assimilation dynamics for *P. acrostichoides* in Connecticut near the northern limit of its distribution (GBIF Secretariat, n.d.) compared with the data of Forget *et al.* (2018) that sampled the same species in North Carolina. Our data show that in the more northern latitude where winters are colder and plants experienced numerous freeze–thaw events and sustained freezing temperatures, *T*_min_ strongly influenced carbon balance, with minimal photosynthesis occurring throughout the winter months. In contrast, Forget *et al.* (2018) found that the bulk of the seasonal carbon gain occurred in the winter when the deciduous canopy was open. However, the populations of *P. acrostichoides* in North Carolina do not consistently reach below freezing temperatures during the winter. Forget *et al.* (2018) conducted a temperature response curve and found that *P. acrostichoides* still achieved a positive carbon gain at 0 °C, which was the lowest ambient temperature measured in their study. At our Connecticut field site, *P. acrostichoides* individuals spend entire days below 0 °C, experiencing a net carbon deficit, as our mean January LRC (Fig. 5) and diurnal net carbon assimilation model **[see ****Supporting Information—Fig. S10g****]** show. Thus, *T*_min_ becomes severely limiting to *P. acrostichoides* winter carbon gain in New England where winter is more harsh compared with previously studied populations in North Carolina (Forget *et al.* 2018), highlighting the potential role of local adaption across the species distribution. Given that *P. acrostichoides* is widespread in eastern North America from Nova Scotia to Florida (GBIF Secretariat, n.d.), future studies should compare winter photosynthesis of *P. acrostichoides* across its whole range.

Evidence from our diurnal net carbon assimilation models shows that days sampled in April, October and November account for 58 % of the total net diurnal carbon assimilation estimates. Thus, these shoulder season months with no overstory deciduous canopy represent an important period of photosynthetic carbon gain for *P. acrostichoides*, along with other wintergreen ferns (Goldblum and Kwit 2012).The wintergreen habit, while conferring the advantage of net positive carbon gain in the shoulder seasons, also comes with the liability of the respiratory costs of keeping foliage alive throughout the winter. The northern limit to the distribution of a wintergreen species would likely be constrained by the amount of time spent in below freezing temperatures, given that net carbon gain is strongly affected by *T*_*min*_.

### Loss of hydraulic function

With an air-seeding threshold pressure of −3.19 MPa and the fact that the lowest frond water potential measured was −1.45 MPa, it is improbable that *P. acrostichoides* experienced drought-induced embolism during our study. Both soil moisture and frond water potential **[see ****Supporting Information—Fig. S7****]** were weak predictors of PLC compared to *T*_min_ (Fig. 6C), suggesting that lost conductivity was driven by temperature and freeze–thaw dynamics. Given the putative relationship between freeze–thaw embolism and conduit diameter (Davis *et al.* 1999; Pittermann and Sperry 2003, 2006), we characterized the stipe tracheid diameter distribution, observing that the average tracheid diameter was 12.8 ± 0.47 µm **[see ****Supporting Information—Fig. S8****]**. The conduit diameter distribution shows good agreement with previously reported data for many gymnosperms and ferns (Sperry *et al.* 1994; Pittermann and Sperry 2003; Pittermann *et al.* 2011; Brodersen *et al.* 2014, 2016). Our data suggest that the majority of the stipe tracheids are below the critical 30–43 µm threshold (Davis *et al.* 1999; Pittermann and Sperry 2003) and would resist most freeze–thaw embolism at less negative xylem pressures. However, it is likely that we observed more embolism than one would expect from freeze–thaw physics alone. Our observed winter embolism was likely due to a combination of repeated freeze–thaw events coincident with reduced water availability while the soil moisture was frozen (Lemoine *et al.* 1999; Améglio *et al.* 2001; Mayr *et al.* 2002, 2003, 2019; Pittermann and Sperry 2006). Frost drought-induced embolism can occur from sun and wind exposure, especially on warm winter days when xylem sap would thaw. Prolonged exposure to dry air leads to cuticular water loss, and transpiration losses from foliage while frond temperature is above freezing while soil water remains frozen would further exacerbate winter water deficits (Lemoine *et al.* 1999; Sparks and Black 2000; Améglio *et al.* 2001; Mayr *et al.* 2002, 2003, 2019; Pittermann and Sperry 2003).

While conduit diameter is a key parameter determining PLC from freeze–thaw embolism, many other factors can govern the formation of winter embolism. Some studies show that *T*_min_ is important for determining the severity of embolism, although the results are mixed depending on the species (Lo Gullo and Salleo 1993; Pockman and Sperry 1997; Ball *et al.* 2006; Pittermann and Sperry 2006). Repeated freezing and thawing cycles can accumulate more embolism than a single freeze–thaw event (Mayr *et al.* 2003, 2019). The thawing rate can influence residual embolism, with a faster thawing rate causing less bubbles to be dissolved into xylem solution (Langan *et al.* 1997; Feild and Brodribb 2001; Pittermann and Sperry 2003). All mechanisms of winter embolism aside, the xylem of *P. acrostichoides* appears to be comparable to that of conifers in terms of its ability to withstand winter embolism and maintain enough functional conductivity (~30–40 %) to preserve photosynthesis into the spring. The blockage of the majority of xylem without substantial decline in gas exchange is not unheard of for ferns; one study shows that cutting one of the two major vascular bundles caused no significant decline in stomatal conductance (Brodersen *et al.* 2012). Together, these results suggest that ferns can be resilient to xylem stressors, with a vasculature that is overbuilt to withstand partial losses of function.

### Hinge zone

While previous studies have examined the functionality of *P. acrostichoides* vascular bundles in the hinge zone with dye experiments (Noodén and Wagner Jr 1997), our hydraulics data are the first to quantitively illustrate that the xylem was functionally conductive through the hinge zone, albeit at reduced conductivities from winter embolism. The hinged sections of stipes had a lower *EI* compared with unhinged sections of stipes (Fig. 7A) because in hinged stipes the parenchymatous cortical cells necrotize around the preserved vascular bundles (Fig.7C). Even though the sclereids in the hyperdermal sterome provide much of the structural support for the stipe, the inner cortical cells reinforce the stipe like a foam core (Gibson 2005, 2012; Vogel 2013; Shah *et al.* 2017), and the stipe buckles once they necrotize. The mechanism for this localized and tissue-specific necrosis in *P. acrostichoides* remains unknown. However, our results corroborate a previous study describing the hinge zone in *P. acrostichoides*, namely that the cortical cells in the hinge zone are dead, loosely attached (i.e. friable), and vascular tissues maintain their structural integrity (Noodén and Wagner Jr 1997). Since our time-lapse movie shows that the timing of the hinge zone formation and subsequent hinging of fronds is related to the onset of freezing temperatures, we cannot completely rule out the possibility that the increase in PLC after the first frost was partially attributed to mechanical damage (i.e. tracheids rupturing) during hinging. While mechanical bending during freezing does not embolize conduits in the angiosperms *Malus* and *Populus* (Christensen-Dalsgaard and Tyree 2013), we do not know how this result applies to fern tracheids. Our anatomical images reveal the vascular bundles are visually intact within the hinge zone (Fig. 7C), leading us to believe that winter embolism was the primary cause of the increase in PLC as temperatures reached below freezing, rather than mechanical damage. The fact that vascular bundles are flexible (Fig. 1) is paramount to their preservation within the hinge zone, as the stipe can bend without breakage of the vasculature.

Previous studies have found that winter leaf prostration and hinging behaviour is advantageous for the energy balance of the leaf in winter (Givnish 1982; Forget *et al.* 2018). We found that fronds that were flat were on average 2.2 °C warmer than fronds that were artificially propped upright, which is consistent with previous studies that examined how leaf temperature changes with leaf angle in *P. acrostichoides* (Givnish 1982; Forget *et al.* 2018). By maintaining a warmer leaf temperature, the flat leaves would be exposed to more light (as opposed to being at a more oblique angle relative to the sun) and remain within a favourable operating temperature range for photosynthesis on a sunny, winter day, while also being protected from frost damage by insulating layers of snow, when present (Tessier 2014). Other proposed physiological explanations for winter leaf prostration include trapping water vapour under the leaf to prevent winter desiccation (Forget *et al.* 2018), mitigation of winter photoinhibition (Forget *et al.* 2018), suppression of growth of neighbouring plants in the spring (Tani and Kudo 2005) and storage ability (e.g. carbon and nutrients) for the next season (Minoletti and Boerner 1993; Tani and Kudo 2003; Goldblum and Kwit 2012). All of these physiological advantages of leaf hinging are made possible by the flexible vascular bundles that do not snap as the frond stipe bends, allowing the hydraulic and photosynthetic systems to remain coupled.

## Conclusion

Here, we provide evidence for a strong coordination between photosynthetic carbon gain, frond stipe hydraulics and leaf temperature for the wintergreen fern *P. acrostichoides*. These anatomical and physiological strategies help to explain the adaptations needed to tolerate winter conditions in the understory. Taking an integrative approach to the wintergreen life strategies of *P. acrostichoides*, we observe that the hinging action and the flexibility of the vascular bundles allow *P. acrostichoides* fronds to survive freezing temperatures by maintaining a warmer leaf temperature, without damaging the vascular bundles. While winter embolism occurs and remains high in overwintered fronds into the spring, photosynthetic rates and efficiency are able to recover to pre-winter levels, and *P. acrostichoides* can jumpstart carbon gain for the year. Our results indicate that the xylem of *P. acrostichoides* is flexible yet robust, as it can tolerate winter embolism and the hinging mechanics, all without damaging the photosynthetic machinery. These wintergreen strategies contribute to the survival of *P. acrostichoides* in northeastern forests.

## Supporting Information

The following additional information is available in the online version of this article—

**Table S1**. Parameters and variable estimates from light response curve models, separated by shade treatment and ambient condition.

**Figure S1**. Comparison of *T*_min_ data from Bradley International Airport Station in Windsor Locks, CT and our own field site weather station in Eastford, CT.

**Figure S2**. *A*_max_ values from the light response curve models show not statistically significant differences between the fronds under the shaded condition (solid line) and ambient condition (two-dash line) (paired *t*-test: *T* = 1.8, DF = 5, *P*-value = 0.13).

**Figure S3**. The linear regression model of modelled *A*_max_ and measured *F*_*v*_*/F*_*m*_ was significant and positive (adjusted *R*^2^ = 0.81, *F* = 34.1, *P* = 0.00064).

**Figure S4**. The linear regression model of *A*_max_ and maximum PPFD of the sampling day was not statistically significant (adjusted *R*^2^ = −0.02, *F* = 0.89, *P* = 0.39).

**Figure S5**. The linear regression model of modelled *A*_max_ and *T*_min_ was significant and positive (adjusted *R*^2^ = 0.44, F = 8.9, *P* = 0.015).

**Figure S6**. The linear regression model of *A*_max_ and frond water potential was not statistically significant (adjusted *R*^2^ = 0.23, *F* = 4.0, *P* = 0.07).

**Figure S7**. (a) The linear regression model for frond water potential and PLC was not statistically significant (adjusted *R*^2^ = 0.09, *F* = 2.1, *P* = 0.176).

**Figure S8**. The tracheid diameter distribution (*n* = 185) shows the presence of many small diameter tracheids, and only a few tracheids measuring 30 μm.

**Figure S9**. Frond temperatures to determine the effect of hinging and lying prostrate on energy balance of the fronds.

**Figure S10**. (a–i) Diurnal carbon assimilation models of *A* (in μmol (CO_2_) m^−2^ s^−1^) for sampling dates throughout the year. Vertical black dashed lines mark the timing of sunrise and sunset for each sampling day. Light grey shading (a and b) represents fronds from the 2016 cohort, while dark grey shading (c–i) represents fronds from the 2017 cohort. (j) Net diurnal carbon assimilation for sampling dates throughout the year. The horizontal grey dashed line is at zero.

**Movie S1.** Time-lapse movie of *P. acrostichoides* leaf prostration from 10 October 2017 to 10 December 2017.

plaa048_suppl_Supplementary_Materials

plaa048_suppl_Supplementary_Data

plaa048_suppl_Supplementary_Video

## Notes

**Form & Function. Chief Editor**: Kate McCulloh
