# Supplementary material for: Seasonal coordination of leaf hydraulics and gas exchange in a wintergreen fern
Source: AoB Plants. 2020 Sep 11;12(6):plaa048. doi: 10.1093/aobpla/plaa048 (PMC7724977; doi:10.1093/aobpla/plaa048)
Supplement: plaa048_suppl_Supplementary_Materials [file plaa048_suppl_supplementary_materials.pdf]

SUPPORTING INFORMATION

Table S1. Parameters and variable estimates from light response curve models, separated by shade treatment and ambient condition. A_{max} is the maximum gross photosynthetic rate in µmol (CO_2) m^{-2} s^{-1}, I_{comp} is the light compensation point in µmol (photons) m^{-2} s^{-1}, I_{max} is the light saturation point in µmol (photons) m^{-2} s^{-1}, R_D is the dark respiration rate in µmol (CO_2) m^{-2} s^{-1}, Φ_{Io} is the quantum yield at $I = 0$ in µmol (CO_2) μmol^{-1} (photons), and $A_{N(I\max)}$ is the net photosynthetic rate at the light saturation point in µmol (CO_2) m^{-2} s^{-1} (Baly, 1935; Lobo et al., 2013). Data for each date of sampling comprised the mean light response curve values (A_N) for two (shade treatment) to four (ambient) *P. acrostichoides* ferns.

**Date of sampling**	***Treatment***	A_{max}	I_{comp}	I_{max}	R_D	Φ_{Io}	$A_{N(I\max)}$
Apr 20, 2017†	Ambient	6.4	17.5	605.0	0.8	0.0548	4.5
	Shade	N/A	N/A	N/A	N/A	N/A	N/A
May 16, 2017†	Ambient	5.0	11.3	468.0	0.6	0.0556	3.6
	Shade	N/A	N/A	N/A	N/A	N/A	N/A
Jun 13, 2017	Ambient	4.7	22.5	442.0	1.0	0.0545	3.0
	Shade	6.2	28.4	437.0	2.0	0.1058	3.4
Jun 27, 2017	Ambient	5.7	10.1	477.0	0.6	0.0717	4.2
	Shade	4.6	2.2	641.0	0.0	0.0191	3.3
Sep 27, 2017‡	Ambient	6.0	9.2	535.0	0.5	0.0635	4.6
	Shade	5.8	15.3	471.0	1.0	0.0770	4.0
Oct 11, 2017§	Ambient	6.9	15.8	602.0	0.9	0.0679	5.0
	Shade	4.9	7.2	409.0	0.5	0.0711	3.7
Nov 8, 2017	Ambient	5.5	3.8	428.0	0.3	0.0867	4.5
	Shade	3.2	4.4	291.0	0.2	0.0555	2.4
Dec 6, 2017	Ambient	4.1	2.7	329.0	0.2	0.0764	3.3
	Shade	3.1	0.0	341.0	0.0	0.0366	2.5
Jan 31, 2018	Ambient	0.5	48.7	65.0	0.2	0.0100	0.0
	Shade	0.6	3.5	74.0	0.0	0.0100	0.3
Apr 12, 2018	Ambient	3.9	12.3	310.0	0.8	0.0772	2.6
	Shade	3.0	4.9	286.0	0.2	0.0488	2.2
May 2, 2018	Ambient	5.6	62.4	561.0	2.0	0.0481	2.7
	Shade	6.4	23.2	555.0	1.3	0.0686	4.2

† 2016 cohort of fronds. All remaining dates sampled from the 2017 cohort of fronds
‡ Shade treatment structures set up in the field
§ First date of sampling the shade treatment

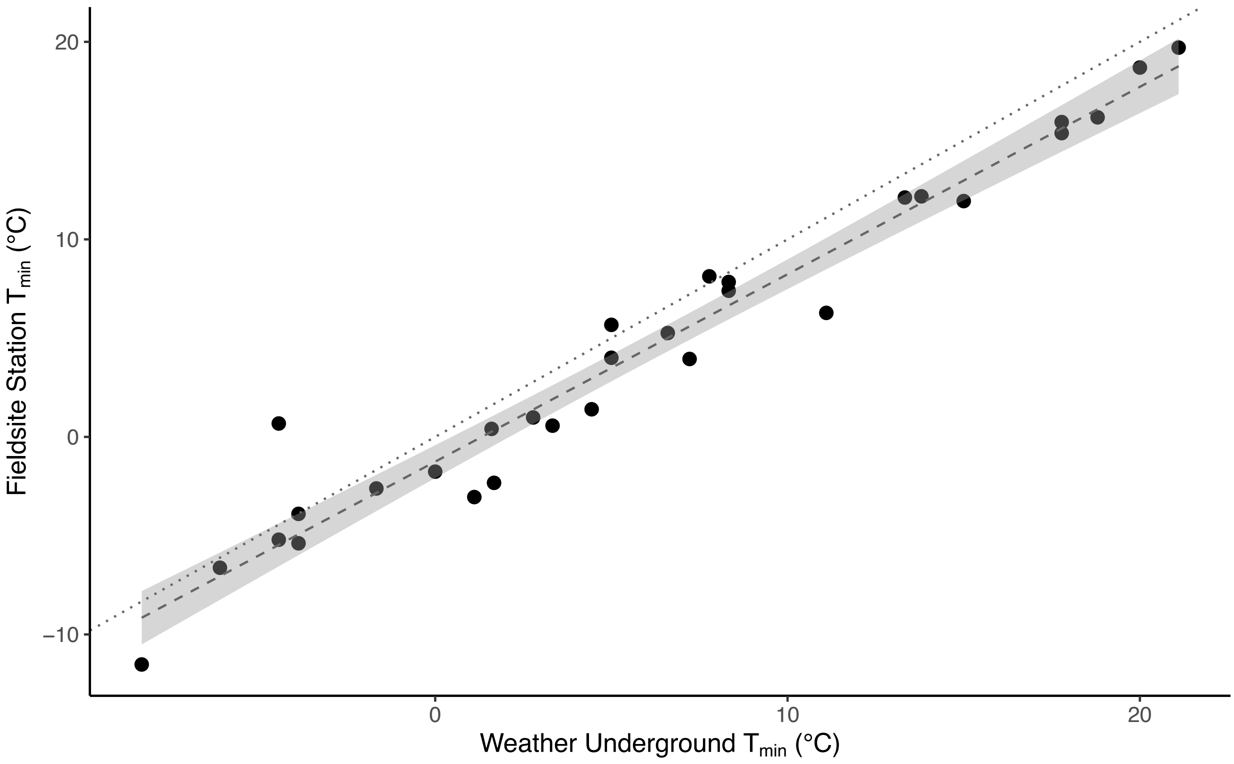

Figure S1. Comparison of T_{min} data from Bradley International Airport Station in Windsor Locks, CT and our own field site weather station in Eastford, CT. The linear regression model was significant (Adjusted R-squared = 0.95, F = 564.2, P = < 2.2e-16). The solid line is the regression line, while the dotted line shows the 1:1 line. All historical data from the Bradley International Airport Station was sourced via Weather Underground (Weather Underground and Company, 2019)

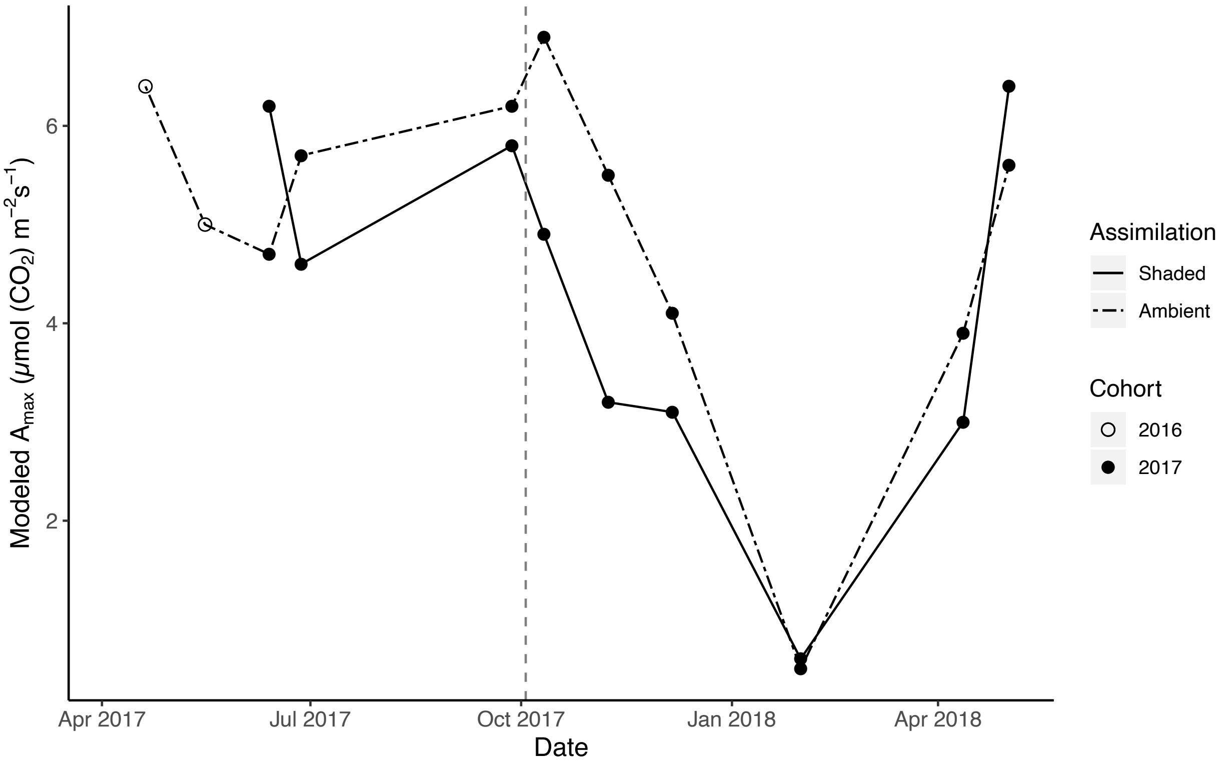

Figure S2. A_{max} values from the light response curve models show not statistically significant differences between the fronds under the shaded condition (solid line) and ambient condition (two-dash line) (paired t-test: T = 1.8, DF = 5, P-value = 0.13). Open circles represent measurements on fronds from the 2016 cohort, while closed circles are fronds from the 2017 cohort. The dashed gray line marks the day that the shade structures were installed at the field site.

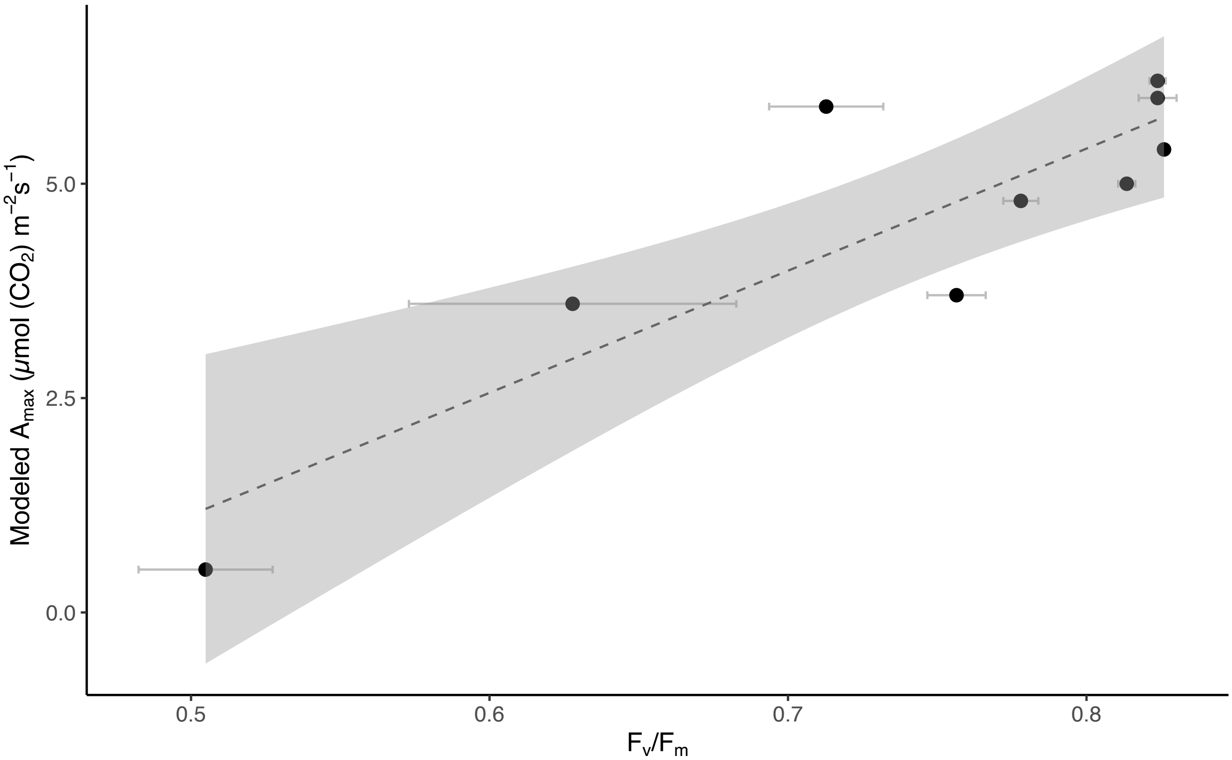

Figure S3. The linear regression model of modeled A_{max} and measured F_v/F_m was significant and positive (Adjusted R-squared = 0.81, F = 34.1, P = 0.00064). Modeled A_{max} values were generated by the Lobo et al. tool (2013) that uses the Baly model (1935) to predict A_{max} from light response curves. All values were generated from fronds that belonged to the 2017 cohort. The gray shaded area around the dashed regression line represents the 95% confidence interval. Gray bars represent standard error of the mean for F_v/F_m.

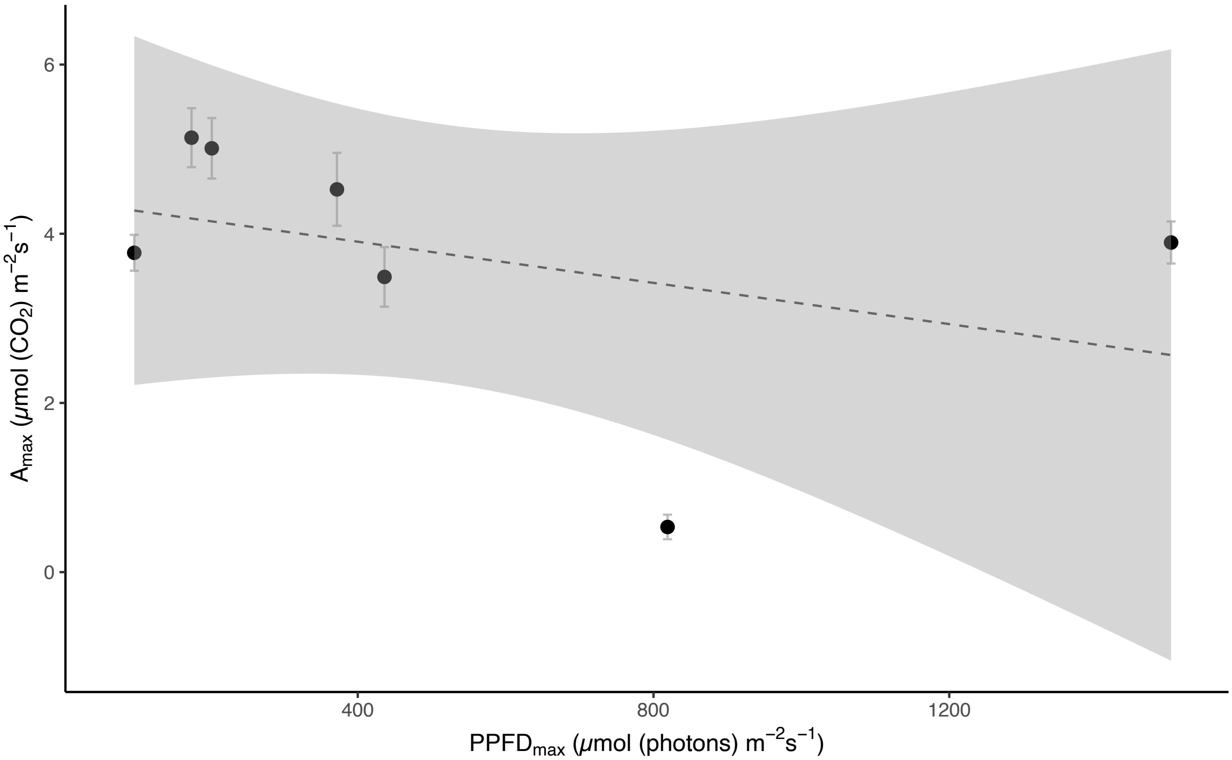

Figure S4. The linear regression model of A_{max} and maximum PPFD of the sampling day was not statistically significant (Adjusted R-squared = -0.02, F = 0.89, P = 0.39). PPFD was only available for fronds from the 2017 cohort, after the installation of the weather station at the field site. The gray shaded area around the dashed regression line represents the 95% confidence interval. Gray bars represent standard error of the mean.

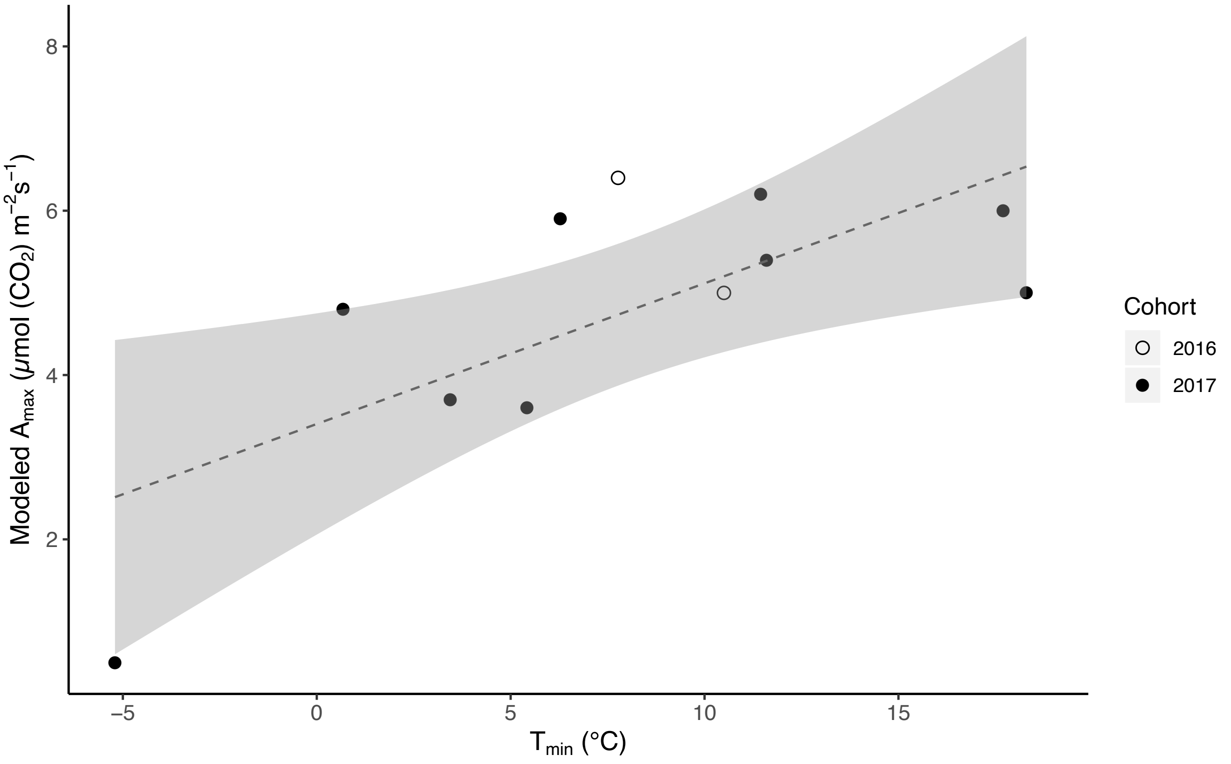

Figure S5. The linear regression model of modeled A_{max} and T_{min} was significant and positive (Adjusted R-squared = 0.44, F = 8.9, P = 0.015). Modeled A_{max} values were generated by the Lobo et al. tool (2013) that uses the Baly model (1935) to predict A_{max} from light response curves. Open circles indicate measurements on fronds from the 2016 cohort, while closed circles are from the 2017 cohort. The gray shaded area around the dashed regression line represents the 95% confidence interval.

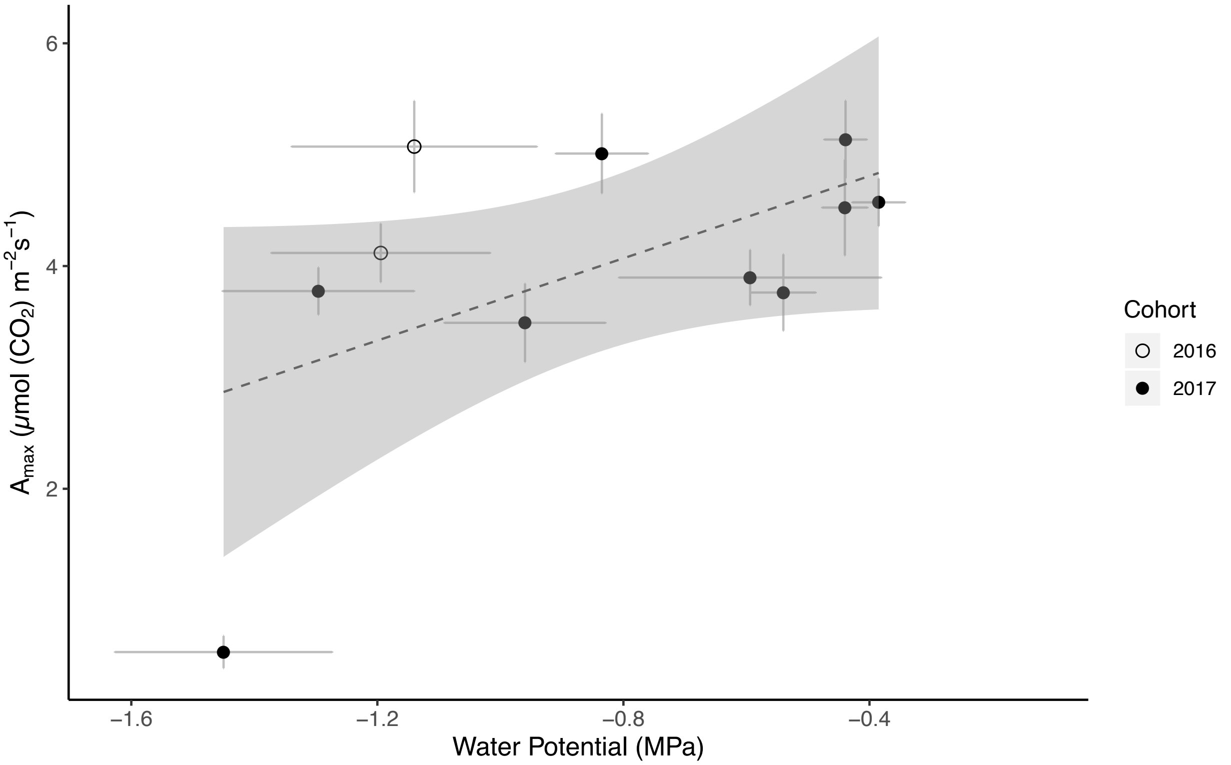

Figure S6. The linear regression model of A_{max} and frond water potential was not statistically significant (Adjusted R-squared = 0.23, F = 4.0, P = 0.07). Open circles indicate measurements on fronds from the 2016 cohort, while closed circles are from the 2017 cohort. The gray shaded area around the dashed regression line represents the 95% confidence interval. Gray bars represent standard error of the mean.

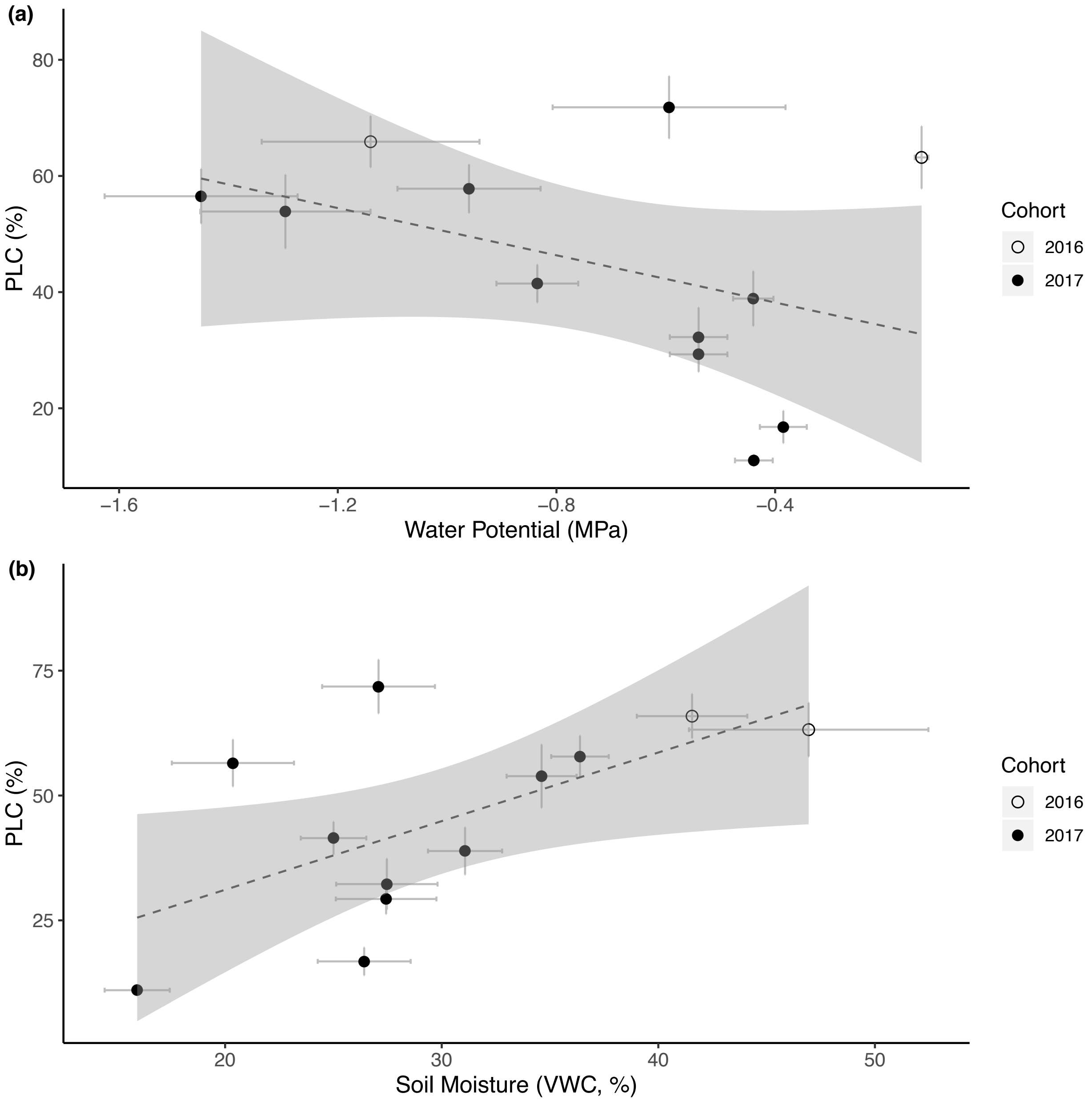

Figure S7. (a) The linear regression model for frond water potential and PLC was not statistically significant (Adjusted R-squared = 0.09, F = 2.1, P = 0.176). (b) The linear regression model for soil moisture and PLC was weak but statistically significant (Adjusted R-squared = 0.31, F = 5.8, P = 0.036). Open circles in (a) and (b) indicate measurements on fronds from the 2016 cohort, while closed circles are from the 2017 cohort. The gray shaded areas around the dashed regression lines represents the 95% confidence interval. Gray bars represent standard error of the mean.

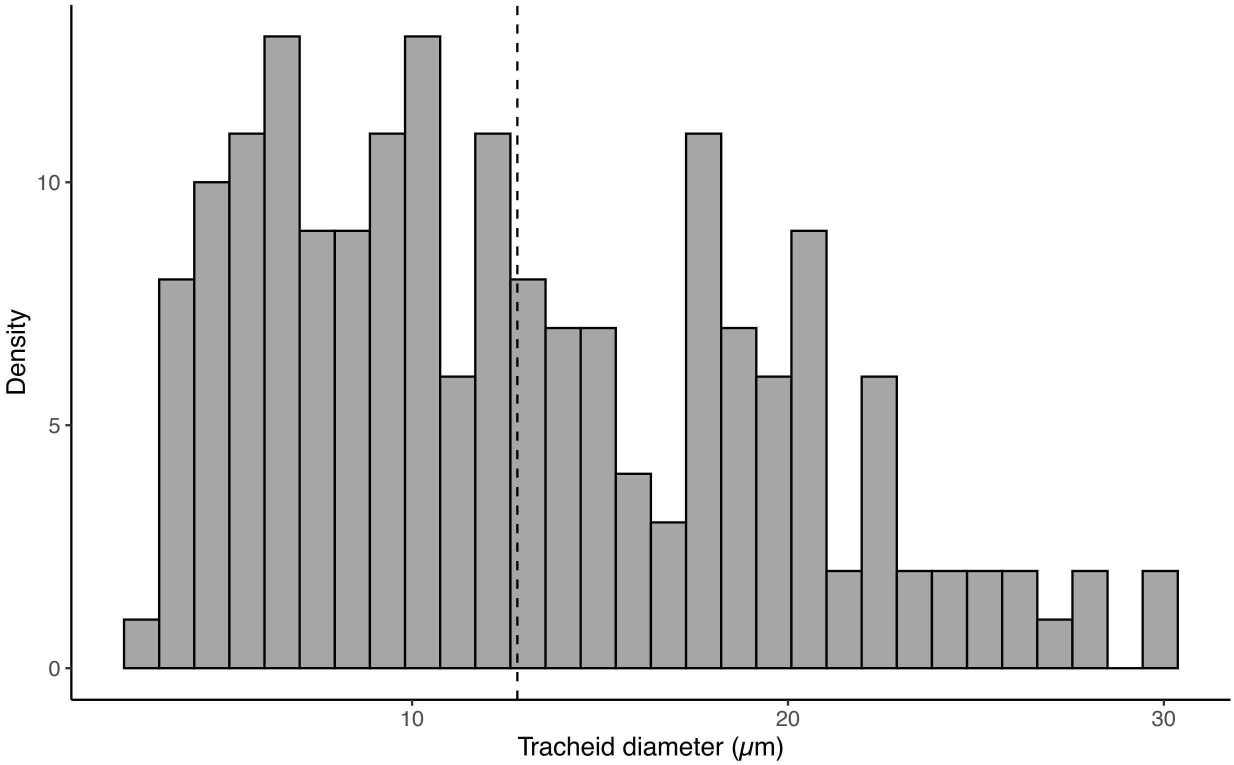

Figure S8. The tracheid diameter distribution (n = 185) shows the presence of many small diameter tracheids, and only a few tracheids measuring 30 μm. The dashed line depicts the mean stipe tracheid diameter of 12.8 ± 0.47 μm. Tracheids from both large and small vascular bundles were measured.

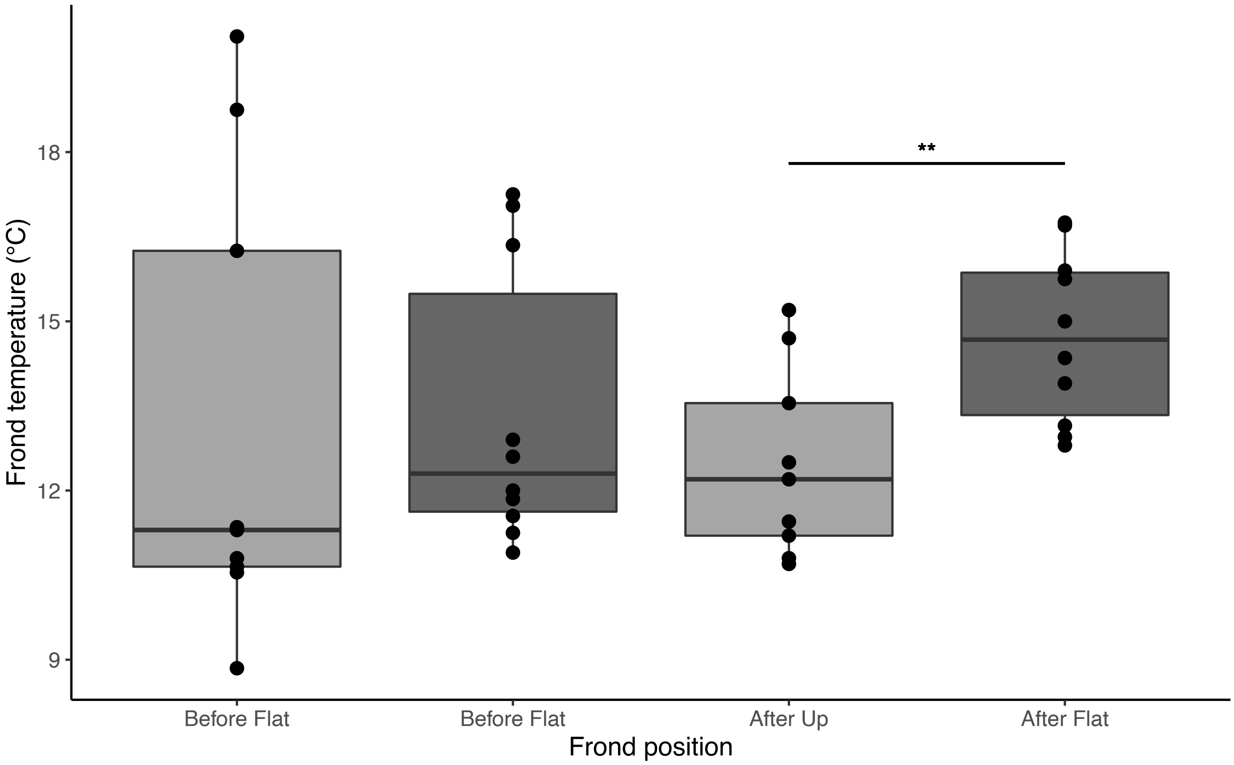

Figure S9. Frond temperatures to determine the effect of hinging and lying prostrate on energy balance of the fronds. We measured two before groups (n = 10 each) where frond position was flat, and those same two groups after where fronds in one group were artificially propped up. The mean leaf temperature of the fronds that remained prostrate was significantly warmer at 14.7°C than the temperature of the fronds that were propped up, at 12.5°C (Welch two-sample t-test: T = 3.06, DF = 16.3, P = 0.007).

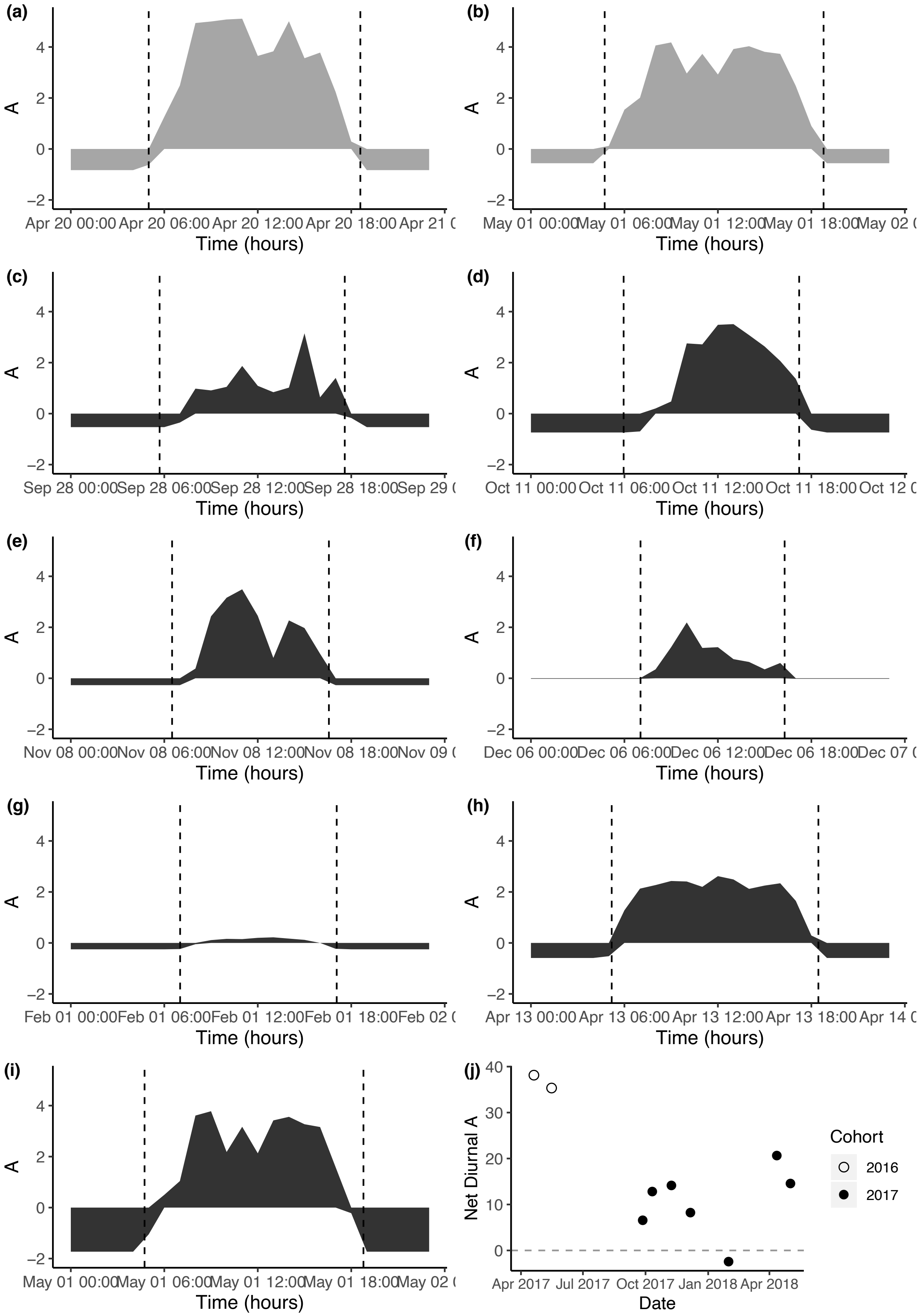

(a)
A
Apr 20 00:00
Apr 20 06:00
Apr 20 12:00
Apr 20 18:00
Time (hours)
(b)
A
May 01 00:00
May 01 06:00
May 01 12:00
May 01 18:00
Time (hours)
(c)
A
Sep 28 00:00
Sep 28 06:00
Sep 28 12:00
Sep 28 18:00
Time (hours)
(d)
A
Oct 11 00:00
Oct 11 06:00
Oct 11 12:00
Oct 11 18:00
Time (hours)
(e)
A
Nov 08 00:00
Nov 08 06:00
Nov 08 12:00
Nov 08 18:00
Time (hours)
(f)
A
Dec 06 00:00
Dec 06 06:00
Dec 06 12:00
Dec 06 18:00
Time (hours)
(g)
A
Feb 01 00:00
Feb 01 06:00
Feb 01 12:00
Feb 01 18:00
Time (hours)
(h)
A
Apr 13 00:00
Apr 13 06:00
Apr 13 12:00
Apr 13 18:00
Time (hours)
(i)
A
May 01 00:00
May 01 06:00
May 01 12:00
May 01 18:00
Time (hours)
(j)
Net Diurnal A
Apr 2017
Jul 2017
Oct 2017
Jan 2018
Apr 2018
Date
Cohort
2016
2017

Figure S10. (a)-(i) Diurnal carbon assimilation models of *A* (in μmol (CO_2) $m^{-2}s^{-1}$) for sampling dates throughout the year. Vertical black dashed lines mark the timing of sunrise and sunset for each sampling day. Light gray shading (a, b) represents fronds from the 2016 cohort, while dark gray shading (c-i) represents fronds from the 2017 cohort. (j) Net diurnal carbon assimilation for sampling dates throughout the year. The horizontal gray dashed line is at zero.

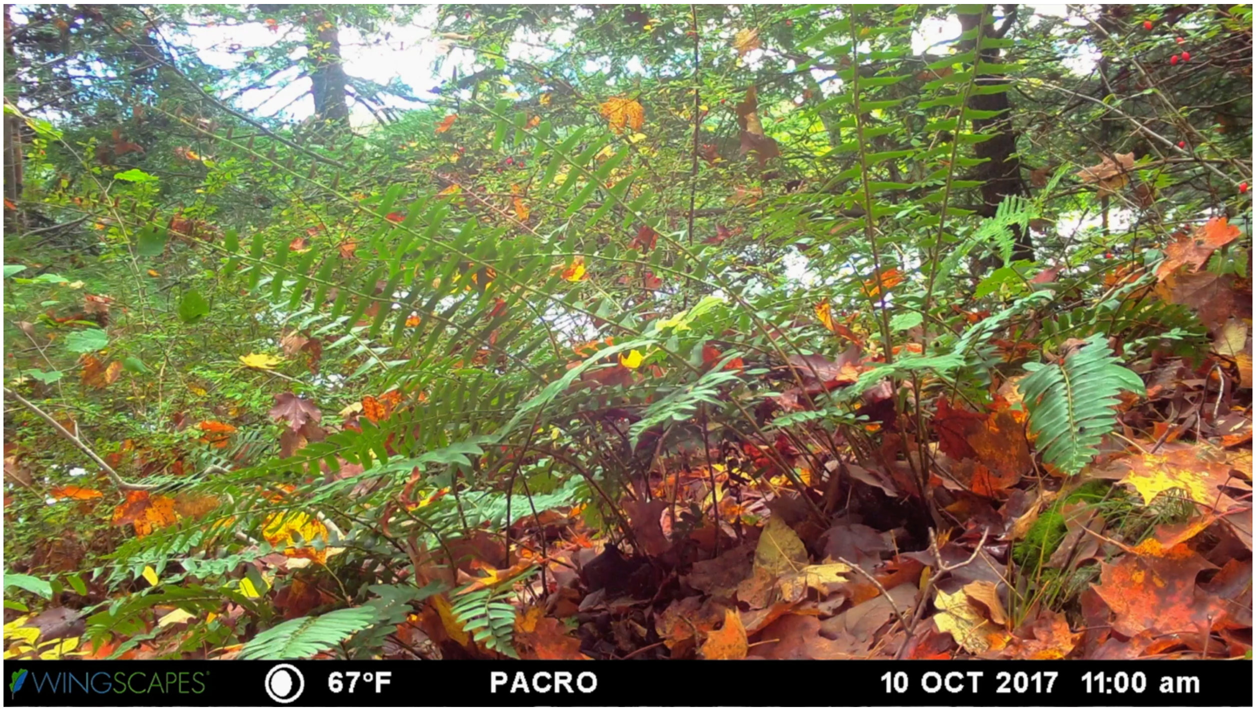

Movie S1. Time-lapse movie of *P. acrostichoides* leaf prostration from October 10, 2017 to December 10, 2017. Still photographs were collected at 11:00 AM daily to document the change in leaf angle during the development of the hinge zone in the stipe. Initial changes in leaf angle occurred around October 29th, with a rapid transition starting on November 9th, and full prostration complete by November 14th.
